# Long-Read Sequencing to Unravel Complex Structural Variants of *CEP78* Leading to Cone-Rod Dystrophy and Hearing Loss

**DOI:** 10.3389/fcell.2021.664317

**Published:** 2021-04-21

**Authors:** Giulia Ascari, Nanna D. Rendtorff, Marieke De Bruyne, Julie De Zaeytijd, Michel Van Lint, Miriam Bauwens, Mattias Van Heetvelde, Gavin Arno, Julie Jacob, David Creytens, Jo Van Dorpe, Thalia Van Laethem, Toon Rosseel, Tim De Pooter, Peter De Rijk, Wouter De Coster, Björn Menten, Alfredo Dueñas Rey, Mojca Strazisar, Mette Bertelsen, Lisbeth Tranebjaerg, Elfride De Baere

**Affiliations:** ^1^Center for Medical Genetics Ghent, Ghent University Hospital, Ghent, Belgium; ^2^Department of Biomolecular Medicine, Ghent University, Ghent, Belgium; ^3^The Kennedy Center, Department of Clinical Genetics, Rigshospitalet, Copenhagen University Hospital, Copenhagen, Denmark; ^4^Department of Ophthalmology, Ghent University Hospital, Ghent, Belgium; ^5^Department of Ophthalmology, Antwerp University Hospital, Antwerp, Belgium; ^6^Great Ormond Street Hospital, London, United Kingdom; ^7^Moorfields Eye Hospital, London, United Kingdom; ^8^UCL Institute of Ophthalmology, London, United Kingdom; ^9^Department of Ophthalmology, University Hospitals Leuven, Leuven, Belgium; ^10^Department of Pathology, Ghent University Hospital, Ghent, Belgium; ^11^Department of Diagnostic Sciences, Ghent University, Ghent, Belgium; ^12^Neuromics Support Facility, VIB Center for Molecular Neurology, VIB, Antwerp, Belgium; ^13^Neuromics Support Facility, Department of Biomedical Sciences, University of Antwerp, Antwerp, Belgium; ^14^Applied and Translational Neurogenomics Group, VIB Center for Molecular Neurology, VIB, Antwerp, Belgium; ^15^Applied and Translational Neurogenomics Group, Department of Biomedical Sciences, University of Antwerp, Antwerp, Belgium; ^16^Department of Ophthalmology, Rigshospitalet-Glostrup, University of Copenhagen, Glostrup, Denmark; ^17^Institute of Clinical Medicine, University of Copenhagen, Copenhagen, Denmark

**Keywords:** CEP78, inherited retinal disease, cone-rod dystrophy with hearing loss, long-read sequencing, structural variants, single-cell gene expression analysis

## Abstract

Inactivating variants as well as a missense variant in the centrosomal *CEP78* gene have been identified in autosomal recessive cone-rod dystrophy with hearing loss (CRDHL), a rare syndromic inherited retinal disease distinct from Usher syndrome. Apart from this, a complex structural variant (SV) implicating *CEP78* has been reported in CRDHL. Here we aimed to expand the genetic architecture of typical CRDHL by the identification of complex SVs of the *CEP78* region and characterization of their underlying mechanisms. Approaches used for the identification of the SVs are shallow whole-genome sequencing (sWGS) combined with quantitative polymerase chain reaction (PCR) and long-range PCR, or ExomeDepth analysis on whole-exome sequencing (WES) data. Targeted or whole-genome nanopore long-read sequencing (LRS) was used to delineate breakpoint junctions at the nucleotide level. For all SVs cases, the effect of the SVs on *CEP78* expression was assessed using quantitative PCR on patient-derived RNA. Apart from two novel canonical *CEP78* splice variants and a frameshifting single-nucleotide variant (SNV), two SVs affecting *CEP78* were identified in three unrelated individuals with CRDHL: a heterozygous total gene deletion of 235 kb and a partial gene deletion of 15 kb in a heterozygous and homozygous state, respectively. Assessment of the molecular consequences of the SVs on patient’s materials displayed a loss-of-function effect. Delineation and characterization of the 15-kb deletion using targeted LRS revealed the previously described complex *CEP78* SV, suggestive of a recurrent genomic rearrangement. A founder haplotype was demonstrated for the latter SV in cases of Belgian and British origin, respectively. The novel 235-kb deletion was delineated using whole-genome LRS. Breakpoint analysis showed microhomology and pointed to a replication-based underlying mechanism. Moreover, data mining of bulk and single-cell human and mouse transcriptional datasets, together with CEP78 immunostaining on human retina, linked the CEP78 expression domain with its phenotypic manifestations. Overall, this study supports that the *CEP78* locus is prone to distinct SVs and that SV analysis should be considered in a genetic workup of CRDHL. Finally, it demonstrated the power of sWGS and both targeted and whole-genome LRS in identifying and characterizing complex SVs in patients with ocular diseases.

## Introduction

During the last years, next-generation sequencing (NGS) techniques mostly relying on short-read sequencing (SRS) have accelerated molecular diagnoses in individuals with inherited retinal diseases (IRDs) (Jespersgaard et al., 2019). IRD is characterized by a tremendous genetic heterogeneity with variants identified in more than 270 genes [RetNet (Retinal Information Network)^1^ ]. Different types of variants can give rise to IRD, both single-nucleotide variants (SNVs) as well as structural variants (SVs), of which copy number variants (CNVs) have been most frequently reported (Khateb et al., 2016; Ellingford et al., 2018; Van Schil et al., 2018; Daiger et al., 2019; Zampaglione et al., 2020). The latter are estimated to contribute to at least 7%–10% of pathogenic alleles in IRD (Khateb et al., 2016; Ellingford et al., 2018; Daiger et al., 2019; Zampaglione et al., 2020). Interestingly, genomic features such as gene size have been shown to correlate with CNV occurrence in IRD genes (Van Schil et al., 2018).

Structural variant detection based on standard molecular karyotyping and on sequencing depth algorithms is capable of detecting only large CNVs, such as deletions and duplications greater than 50 kb in size, whereas cryptic SVs, such as smaller CNVs, copy neutral, and complex rearrangements, may be missed (Lindstrand et al., 2019). Nowadays, it is possible to detect approximately 27,000 SVs (>50 bp) per human genome only using a combination of technologies, where the majority of these SVs is located in the non-coding part of the genome and missed using exome-based approaches (Belkadi et al., 2015; Chaisson et al., 2019; Mahmoud et al., 2019). Whole-genome sequencing (WGS) proved to be particularly powerful to detect SVs, variants in GC-rich regions, and variants in non-coding regulatory regions (Nishiguchi et al., 2013; Ellingford et al., 2016; Carss et al., 2017; de Bruijn et al., 2020). While predictions are indicating that at least 48% of deletions and 83% of insertions are routinely missed by short-read-calling algorithms (Eichler, 2019), long-read sequencing (LRS) is particularly valuable for detecting SVs, as the long reads provide the necessary context to call and resolve SVs, regardless of their sequence composition (De Coster and Van Broeckhoven, 2019).

One of the more recently identified disease genes that contribute to the genetic heterogeneity of IRD is the centrosomal gene *CEP78* (MIM# 617110), in which several types of variants, inactivating sequence variants as well as a unique missense variant have been found in autosomal recessive cone-rod dystrophy with hearing loss (CRDHL; MIM# 617236), a recognizable phenotype distinct from Usher syndrome (Fu et al., 2016; Namburi et al., 2016; Nikopoulos et al., 2016; Ascari et al., 2020). Apart from CRDHL, sperm abnormalities causing infertility have been reported in two unrelated affected males (Ascari et al., 2020). Functional studies pointed to a loss-of-function effect with decreased amounts of protein, normal subcellular localization, and elongated primary cilia in patients’ cells (Namburi et al., 2016; Nikopoulos et al., 2016; Ascari et al., 2020). Interestingly, a complex SV implicating *CEP78* has been reported in one individual with CRDHL, being a homozygous deletion–inversion–deletion overlapping *CEP78* (Sanchis-Juan et al., 2018). CEP78 localizes to the mature centrioles (Brunk et al., 2016), which are the main components of the centrosomes, key microtubule-organizing hubs in eukaryotic cells, with the mother centriole acting as the basal body during cilia formation (Gönczy and Hatzopoulos, 2019). Centrioles duplicate once per cell cycle, and irregularities in their structure, or number, are associated with several diseases including cancer or ciliopathies (Nigg and Raff, 2009; Gönczy, 2015).

Here, we report *CEP78* SVs and SNVs in four unrelated CRDHL families of Belgian, Danish, and Turkish origin. Interestingly, two distinct SVs were identified in three of the four CRDHL families using a combination of NGS technologies. Targeted or whole-genome nanopore LRS was used to delineate breakpoint junctions at the nucleotide level. For the smallest SV, a founder effect was shown. Overall, this study supports that the *CEP78* locus is prone to distinct SVs and emphasizes the importance of SV analysis in the genetic workup of CRDHL, leveraging the power of shallow whole-genome sequencing (sWGS) and both targeted and whole-genome LRS. Furthermore, data mining of bulk and single-cell (sc) transcriptional datasets, in combination with CEP78 immunostaining, displayed a CEP78 expression domain in agreement with the phenotypic manifestations of CRDHL.

## Materials and Methods

### Ethics Statement

This study followed the tenets of the Declaration of Helsinki, and ethical approval was given by the local ethics committee (Ghent University Hospital, EC UZG 2017/1540). All individuals involved gave their informed consent prior to inclusion in this study.

### Phenotypic Evaluation

Affected individuals were subjected to ophthalmologic evaluation including best-corrected visual acuity measurement, funduscopy, visual field assessment, infrared and blue light reflectance and autofluorescence imaging, spectral-domain optical coherence tomography, and electroretinography, when possible. In addition, an audiological assessment was performed before or after the molecular diagnosis.

### Whole-Exome Sequencing and Variant Validation

Genomic DNA (gDNA) was extracted from leukocytes according the manufacturer’s guidelines. Whole-exome sequencing (WES) was performed using SureSelectXT Human All Exon V6 enrichment (Agilent Technologies) and HiSeq 3000 sequencing (Illumina) (F1 and F4), or SureSelectXT Low Input Human All Exon V7 kit (Agilent Technologies) and NovaSeq 6000 sequencing (Illumina) (F3). Reads were aligned to the human hg38 reference genome with BWA (v0.7.15) (Li and Durbin, 2009). SNVs and small insertions and deletions were detected with the GATK HaplotypeCaller (v3.8^2^). The VCF (variant call format) files were annotated with the Ensembl Variant Effect Predictor (release 95) and the dbNSFP (v3.4a) and dbscSNV (v1.1) databases. Variants were scored heterozygous or homozygous and were assessed with our in-house variant filtering and visualization tool. A selection of 272 and 275 RetNet genes was assessed, respectively (versions 3 and 4 of the RetNet panel). Nucleotide numbering was done following HGVS guidelines^3^ with nucleotide “A” of the ATG as “c.1.” Classification of variants was based on the ACMG and ACGS guidelines with adaptations (Richards et al., 2015; Nykamp et al., 2017; Abou Tayoun et al., 2018; Ellard et al., 2019). CNVs were assessed using ExomeDepth (Plagnol et al., 2012) (v1.1.10) and Pindel (Ye et al., 2009). Pathogenic (likely) variants were confirmed *via* Sanger sequencing. Sanger sequencing was performed using an ABI 3730xl DNA Analyzer (Applied Biosystems) with the BigDye Terminator Cycle Sequencing Kit (Applied Biosystems).

For F2, II:1 gene panel sequencing of 13 genes [*ABHD12*, *ADGRV1* (*GPR98*), *CDH23*, *CIB2*, *CLRN1*, *DFNB31* (*WHRN*), *HARS1*, *MYO7A*, *PCDH15*, *PDZD7*, *USH1C*, *USH1G*, and *USH2A*] associated with Usher syndrome was performed using a HaloPlex custom design created by use of Agilent SureDesign and included exons and 50 bp of the intron at each intron–exon boundary. The enrichment library from patient DNA was sequenced on a MiSeq sequencer (Illumina). FASTQ files were analyzed using SureCall v.3.0.1.4 (Agilent Technologies) using default settings. Whole-exome trio sequencing was performed for F2, II:1 and her parents. Library preparation was done using the Ion AmpliSeq exome kit (Thermo Fisher), and libraries were sequenced using the IonProton system (Thermo Fisher). Base calling, read alignment, and variant calling were performed using the Torrent Suite including the Torrent Variant Caller (Thermo Fisher). VarSeq (GoldenHelix) was used for annotation and filtering of the variants. Confirmation of the identified *CEP78* variant was performed *via* Sanger sequencing using NM_001098802.2 as reference sequence.

### Shallow WGS

Shallow whole-genome sequencing was performed using the Hiseq3000 (Illumina), starting from 200 ng of gDNA. For library construction the NEXTflex Rapid DNA Sequencing kit (Bio Scientific) was used. Pipetting steps were automated using a Hamilton Star robot (Hamilton). Library concentrations were measured by the Qubit High-Sensitivity kit (Thermo Fisher Scientific), and equimolar concentrations were pooled before sequencing. The minimal number of mapped reads was set at 50 million. Copy number analysis was performed with WisecondorX and further visualized with the ViVar platform (Sante et al., 2014; Raman et al., 2019).

### Quantitative and Long-Range Polymerase Chain Reaction for Deletion Confirmation and Delineation

Copy number variants were confirmed by quantitative polymerase chain reaction (qPCR), and primers were designed in coding exons or intronic regions as previously described (D’haene et al., 2010). Assays were prepared using SsoAdvanced Universal SYBR Green Supermix (Bio-Rad Laboratories) and run on LightCycler 480 System (Roche). Data were analyzed with qbase + software (Biogazelle). *ZNF80* and *GPR15* were used as reference genes. Subsequent to iterative qPCRs (long-range), PCR was performed to obtain junction deletion products using Phusion High-Fidelity PCR kit (New England Biolabs) and visualized on 1% UltraPure Agarose (Thermo Fisher Scientific) gels. Primer design was done using Primer3Plus. Primers sequences are listed in Supplementary Table 1.

### Targeted LRS

Amplified (long-range) PCR product was quality checked using DropSense (Trinean), Qubit (ThermoFisher), and Fragment Analyzer (Agilent), using DNF-492 Large Fragment analysis kit (Agilent). A library was constructed according to the Ligation sequencing protocol (SQK-LSK109, Oxford Nanopore Technologies, ONT; GDE_9063_v109_revU_14Aug2019) with minor adaptations. DNA repair and end-prep using ONT consumables (SQK-LSK109) and NEBNext FFPE DNA repair mix and NEBNext Ultra II End repair/dA tailing Module (M6630, E7546 both from New England Biolabs) started with 100 fmol of the PCR amplicon, with extended enzymatic incubation times, 30 min at 20°C and 30 min at 65°C. Repaired and end-prepped amplicon was Ampure XP (Beckman Coulter) cleaned up for increased adaptor ligation efficiency, using a ratio of 1:1 (vol/vol) and extended incubation (10 min on Hulamixer). After bead cleanup, the pellet was eluted in 32 μL Nuclease Free Water, of which 30 μL was used for adapter ligation. Adapter ligation using SQK-LSK109, ONT, and NEBNext Quick Ligation Module (E6056, New England Biolabs) was performed according to the protocol with extension of the ligation incubation to 30 min at room temperature. The ligation reaction was cleaned up using Ampure XP (Beckman Coulter) in vol/vol ratio of 0.4 with extended incubation (10 min on Hulamixer). A purified pellet was eluted in 10 μL of MilliQ water with the final yield of library calculated using concentration and size information (F1, II:1 yield = 508.2 ng/72.6 fmol and F3, II:1 yield = 462 ng/61 fmol). Ten fmol (64 ng for F1, II:1) and 20 fmol (151.5 ng for F3, II:1) amount of the library was loaded and sequenced on MinION using Flongle Flowcell (both Oxford Nanopore Technologies) with, respectively, 85 (F1, II:1) and 47 (F3, II:1) number of pores sequencing after loading of the library. Sequencing was complete in 24 h and generated in total 1.08 Gb (F1, II:1) and 827.9 Mb (F3, II:1) of data, equaling 172.75 K (F1, II:1) and 140.99 K (F3, II:1) reads with an estimated N50 of 10.55 kb (F1, II:1) and 10.7 kb (F3, II:1).

### Whole-Genome LRS

Extracted DNA was checked for concentration, purity, and integrity using DropSense (Trinean), Qubit (ThermoFisher), and Fragment Analyzer (Agilent), using 464 High Sensitivity Large Fragment 50-Kb kit (Agilent). The sample was sheared using Mega3 (Diagenode) to the final average size of the peak 21,015 bp (smear analysis 25,230 bp). Short fragments were eliminated using SRE XS (Circulomics). Sheared and size-selected DNA sample was used in library prep following the protocol gDNA by Ligation (SQK-LSK109, GDE_9063_v109_revU_14Aug2019, Oxford Nanopore Technologies) with minor adaptations. DNA repair and end-prep using ONT consumables (SQK-LSK109) and NEBNext FFPE DNA repair mix and NEBNext Ultra II End repair/dA tailing Module (M6630, E7546 both New England Biolabs) started with 182 fmol of the PCR amplicon, with extended enzymatic incubation times, 30 min at 20°C and 30 min at 65°C. Repaired and end-prepped amplicon was Ampure XP (Beckman Coulter) cleaned up for increased adaptor ligation efficiency, using a ratio of 1:1 (vol/vol) and extended incubation (10 min on Hulamixer). After bead cleanup, the pellet was eluted in 63 μL nuclease-free water, of which 60 μL was used for adapter ligation. Adapter ligation using SQK-LSK109, ONT, and NEBNext Quick Ligation Module (E6056, New England Biolabs) was performed according to the protocol with extension of the ligation incubation to 30 min at room temperature. The ligation reaction was cleaned up using Ampure XP (Beckman Coulter) in vol/vol ratio of 0.4 with extended incubation (10 min on Hulamixer). A purified pellet was eluted in 40 μL of MilliQ water with final yield of library calculated using concentration and size information (yield = 1,627.4 ng/105.6 fmol). Thirty femtomoles of the final library prep was loaded onto PromethION Flow cells (Oxford Nanopore Technologies). In order to generate enough data, the sample was loaded on three flow cells (FCs). Runs started with 6,844 (FC1), 5,835 (FC2), and 6,314 (FC3) sequenceable pores and ran for 72 h, generating in total 32.55 Gb (FC1), 36.18 Gb (FC2), and 40.99 Gb (FC3) of data, equaling 2.12 M (FC1), 2.39 M (FC2), and 2.68 M (FC3) reads with an estimated N50 of 27.86 kb (FC1), 28.76 kb (FC2), and 28.96 kb (FC3).

### Data Analysis LRS

Base calling of the Nanopore data was performed using the Guppy base caller (v4.0.9 + 92ae093). Further analysis was performed using a pipeline integrated in GenomeComb (0.101.0) (Reumers et al., 2012). Reads were aligned to the hg38 genome reference (Schneider et al., 2017) using minimap2 (2.17-r941) (Li, 2018), and the resulting SAM file sorted and converted to BAM using Samtools (1.10) (Li et al., 2009). SVs were called using Sniffles (1.0.11) (Sedlazeck et al., 2018). The region of interest was filtered out and scanned for well-supported SVs using GenomeComb (Reumers et al., 2012).

### Expression Analysis on Patient’s Material and Splicing Assessment

For quantitative reverse transcription–PCR (RT-PCR), total RNA was extracted from short-term cultured lymphocytes or fibroblasts using MagCore according the manufacturer’s guidelines. cDNA was synthesized with the iScript cDNA Synthesis Kit (Bio-Rad Laboratories). For each cDNA sample, assays were prepared using SsoAdvanced Universal SYBR Green Supermix (Bio-Rad Laboratories) and run on LightCycler 480 System (Roche). Data were analyzed with qbase + and normalized to the *YWHAZ* and *HMBS* or *SDHA* genes. Primers were designed using Primer3plus, and sequences are listed in Supplementary Table 1. For non-quantitative RT-PCR, cDNA was synthesized using SuperScript IV Reverse Transcriptase kit (Thermo Fisher) and underwent standard PCR, loaded on 2% UltraPure Agarose gel, and Sanger sequenced.

### Haplotype Analysis

In total, 18 single-nucleotide polymorphisms (SNPs) (dbSNP, build 151) were selected for haplotype reconstruction in the upstream and downstream areas flanking *CEP78*. SNPs were Sanger sequenced according to the standard procedures (see above). PCR primers were designed with Primer3Plus, and sequences can be found in Supplementary Table 2.

### *CEP78* Expression in Mouse and Human Retina and Inner Ear

#### Data Mining in Single-Cell Retinal and Cochlear Transcriptional Datasets

Human adult retinal and murine P1 cochlear sc transcriptional datasets were mined for evaluating *CEP78* expression at the sc level. Expression matrices derived from pooling three donor neural retinal (Cowan et al., 2020) and four cochlear samples (Kolla et al., 2020) were retrieved and processed separately using SCANPY (v1.4.6) (Wolf et al., 2018). Preprocessing and quality control were conducted to remove outlier cells. Briefly, we considered only genes with counts of at least three cells and filtered out cells that had unique feature counts <200 or >2,500 and/or that expressed >5% mitochondrial counts. The data were then total-count normalized, logarithmized, filtered for highly variable features, and scaled to unit variance. After quality control preprocessing, a total of 19,768 retinal and 11,332 cochlear cells were kept for subsequent dimensionality reduction, embedding, and clustering. Markers associated with major neural retina and cochlear cell populations were used to assess *CEP78* expression at the sc level.

#### Data Mining in Bulk Retinal and Cochlear Transcriptional Datasets

Retina— Expression levels by transcripts per million (TPMs) were retrieved from postmortem retina samples characterized in Ratnapriya et al. (2019). From 453 samples that passed quality control, we only considered donor retinas that showed no age-related macular degeneration progression (*n* = 102) to avoid confounding variables in downstream analyses. To remove potential noise, 20% of genes with the lowest mean expression across all samples were filtered out. TPM values were then filtered for a set of candidate genes, which included all genes reported to cause IRD (RetNet) and ciliary genes (SCGSv1) (van Dam et al., 2013). A total of 519 genes were eventually considered. Before evaluating correlations in the expression of the candidate genes, the set was subjected to a variance-stabilizing transformation to correct for mean-variance dependency (Zwiener et al., 2014). We then examined the expression of *CEP78* and several ciliary genes listed in the top 10 of the Human Gene Connectome (HGC) (Itan et al., 2013) (*SCLT1*, *MKS1*, *CEP57*, *CEP76*, *CEP135*, *CEP152*, *CEP63*, *CEP164*, *OFD1*, and *CEP250*). Spearman correlations were computed along with pairwise *p* values adjusted for multiple comparisons (Holm method). A correlogram was then generated for visualization.

Cochlea— We retrieved paired-end FASTQ files (GSE111348) derived from adult (P28-P32) mouse inner (IHC) and outer (OHC) cochlear hair cells (∼1,000 cells per sample; *n* = 4 and 6 for IHC and OHC, respectively) (Li et al., 2018). Transcripts were quantified through pseudoalignment by Kallisto (v.0.46.1), for which default parameters were used for both index build and transcript quantification (Bray et al., 2016). Additionally, data generated by Schrauwen et al. (2016) were used to retrieve expression values of *CEP78* in adult human cochlea and components of the vestibular labyrinth.

### CEP78 Immunostaining on Human Retina

Human retina used for immunohistochemistry was fixed in 10% neutral buffered formaldehyde and embedded in paraffin. Staining for CEP78 was performed on 3-μm-thick sections using an automatic immunostainer (BenchMark Ultra, Ventana Medical Systems, Tucson, AZ, United States). The rabbit polyclonal antibody anti-CEP78 (1:100; LN2004459, LabNed) was used, and visualization was achieved with the OptiView Amplification Kit (Ventana Medical Systems). Heat-induced epitope retrieval was performed using Cell Conditioning 2 (Ventana Medical Systems).

## Results

**FIGURE 1:**
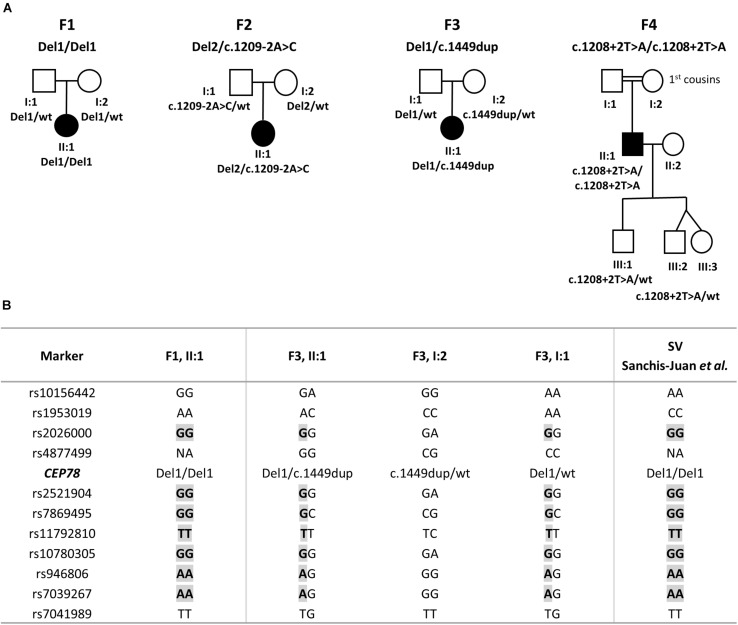
Pedigrees segregating the *CEP78* structural and sequence variants and haplotype reconstruction for the shared SV. **(A)** F1, II:1 is homozygous for a complex deletion–inversion–deletion involving exons 1–5 of *CEP78*. F2, II:1 is compound heterozygous for a deletion of 235 kb overlapping *CEP78* and *PSAT1* and for splice site variant c.1209-2A>C. F3, II:1 is compound heterozygous for the same deletion–inversion–deletion of F1, II:1 and the c.1449dup variant in *CEP78*. F4, II:1 is homozygous for splice site variant c.1208+2T>A. Extended F4 pedigree available in Supplementary Clinical Data 2. **(B)** Genotyping of 18 flanking single-nucleotide polymorphisms (SNPs) revealed a common haplotype of 1.9 Mb, between the individuals carrying the deletion–inversion–deletion [F1, F3 and the case originally described by Sanchis-Juan et al. (2018)]. Extended version available in Supplementary Table 5. Abbreviations: Del, deletion (or deletion–inversion–deletion); wt, wild type.

The CRDHL families included in this study are of Belgian (F1 and F3), Danish (F2), and Turkish origin (F4). Consanguinity was reported for F4. Pedigrees are represented in Figure 1.

### CNV Analysis on WES Data, sWGS, Long-Range PCR, and Targeted LRS in F1

**FIGURE 2:**
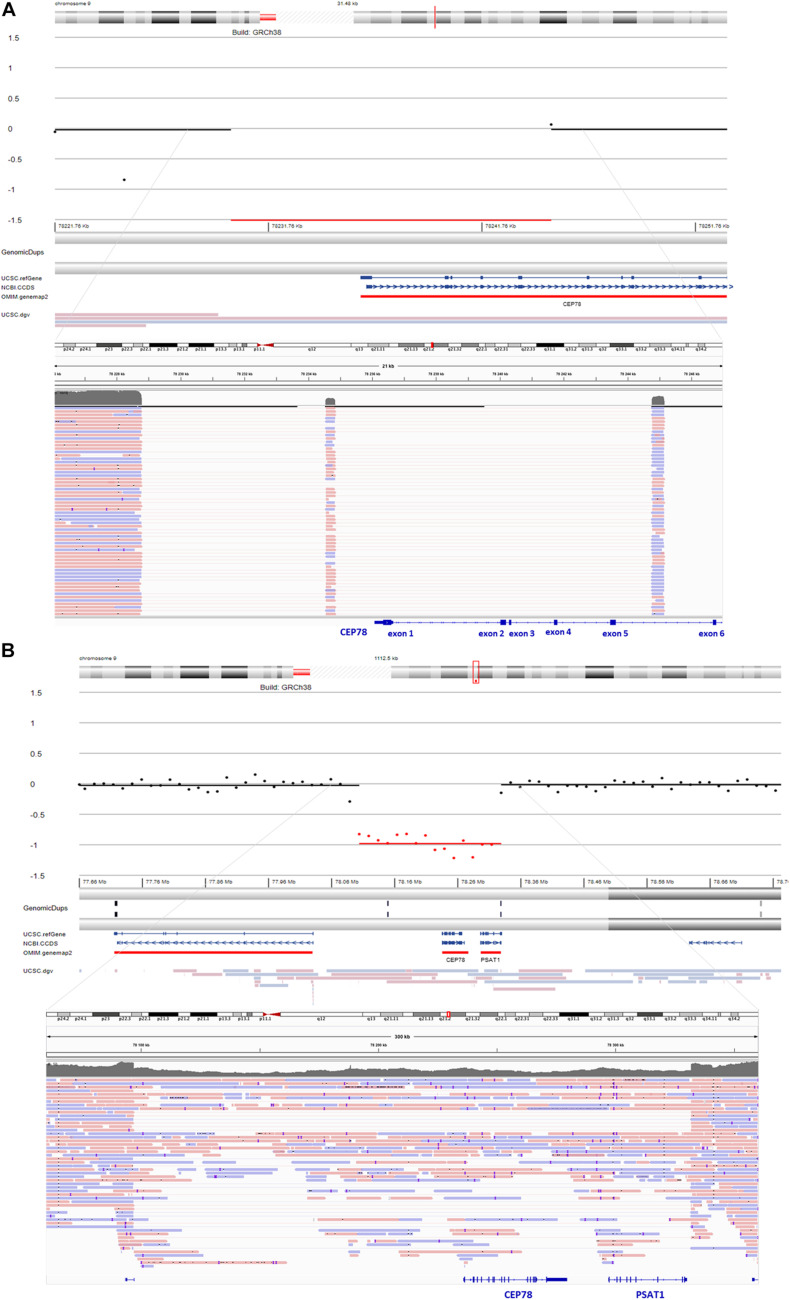
sWGS output and integrative genomics viewer (IGV) view of the structural variants (SVs) detected *via* long-read sequencing (LRS) in F1 and F2. **(A)** sWGS in F1, II:1 revealed a homozygous deletion of the region spanning exons 1–5 of *CEP78* (top). Subsequent delineation of the SV *via* targeted LRS of the junction product identified a complex deletion–inversion–deletion (bottom). The left breakpoint is located at (hg38) chr9:78228782, whereas the right breakpoint is located at chr9:78244762. The inverted segment spans chr9:78234546–78234844 [nearby an L1ME3Cz repetitive element (chr9:78234521–78234902)]. The SV overlaps with a previously described SV affecting *CEP78* (Sanchis-Juan et al., 2018). **(B)** sWGS in F2, II:1 identified a heterozygous deletion spanning the entire *CEP78* and *PSAT1* genes (top). Final delineation was obtained *via* whole-genome LRS, coordinates are crossing chr9: 78096930–78331887 and cover 235 kb (bottom).

For F1, II:1 CNV analysis on WES data using ExomeDepth revealed a potential homozygous deletion overlapping *CEP78* [initial coordinates (hg38) chr9: g.78236351–78243636]. Subsequent sWGS confirmed the homozygous deletion spanning exons 1–5 of *CEP78* [new coordinates (hg38) chr9:g.78230001–78245000], covering a region of 15–20 kb. The sWGS output is available in Supplementary Table 3. The deletion could be refined up to ∼12 kb using iterative qPCRs, and a junction product could be obtained *via* long-range PCR (Supplementary Figure 1). Targeted LRS on the long-range PCR amplicon allowed final delineation, identifying a complex deletion–inversion–deletion (Figure 2). The left breakpoint is located at (hg38) chr9:78228782, whereas the right breakpoint at chr9:78244762. The inverted segment spans chr9:78234546–78234844 [nearby an L1ME3Cz repetitive element (chr9:78234521–78234902)]. The SV overlaps with the one previously described in a British patient by Sanchis-Juan et al. (2018), delineated using microarray and Sanger sequencing (Sanchis-Juan et al., 2018). A smaller deletion, overlapping exons 2 and 3 in *CEP78*, is reported in gnomAD SV (DEL_9_103019; gnomAD SVs v2.1). Segregation analysis was performed *via* qPCR and confirmed both parents as heterozygous carriers of the deletion. Expression analysis of *CEP78* mRNA on available lymphocytes (F1, II:1) and three controls showed complete loss of *CEP78* expression due to the homozygous deletion (Supplementary Figure 2).

### Segregation Analysis, sWGS, and Whole-Genome LRS in F2

For F2, II:1, initial targeted gene panel sequencing was negative, as it was carried out before the *CEP78* gene had been associated with CRDHL. Thus, trio WES was performed for the proband and revealed a novel heterozygous splice variant c.1209-2A>C p.(?) (NM_001098802.2) in *CEP78*. The c.1209-2A>C variant in *CEP78* was confirmed *via* Sanger sequencing, where the proband appeared homozygous, the father heterozygous and the mother wild-type (Supplementary Figure 3). No additional family members have been tested. The splice variant is located in the acceptor splice site of intron 9, reported in dbSNP (rs778035330) with a frequency of 0.00002628 (gnomAD), and predictions suggest a skip of exon 10. It is predicted to disrupt the consensus splice site and likely results in an absent or disrupted protein product and loss of function. Subsequent RT-PCR confirmed the skip of exon 10 (Supplementary Figure 3). Population frequencies and *in silico* predictions of the *CEP78* splice variant are listed in Supplementary Table 4 and Supplementary Figure 4. To our knowledge, this variant has not been reported in literature, but is reported as likely pathogenic in one case in ClinVar (ID 851351). Segregation analysis suggested a heterozygous genomic deletion of the *CEP78* region in F2, II:1, *in trans* with the splice variant. The heterozygous deletion was confirmed by sWGS, showing an interval of 225–240 kb spanning the entire *CEP78* and *PSAT1* genes (chr9: g.78105001–78330000). The sWGS output is available in Supplementary Table 3. The final delineation was obtained *via* whole-genome LRS allowing the identification of a heterozygous (13 reference sequence reads, 21 deletion reads) deletion spanning the region chr9:78096930–78331887 and covering 235 kb (Figure 2). Breakpoint junction analysis highlighted microhomology (Supplementary Figure 5). Expression analysis of *CEP78* mRNA on available fibroblasts (F2, II:1) and two controls showed loss of *CEP78* expression due to the *CEP78* genotype (Supplementary Figure 2).

### CNV Analysis on WES Data, Long-Range PCR, and Targeted LRS in F3

**FIGURE 3:**
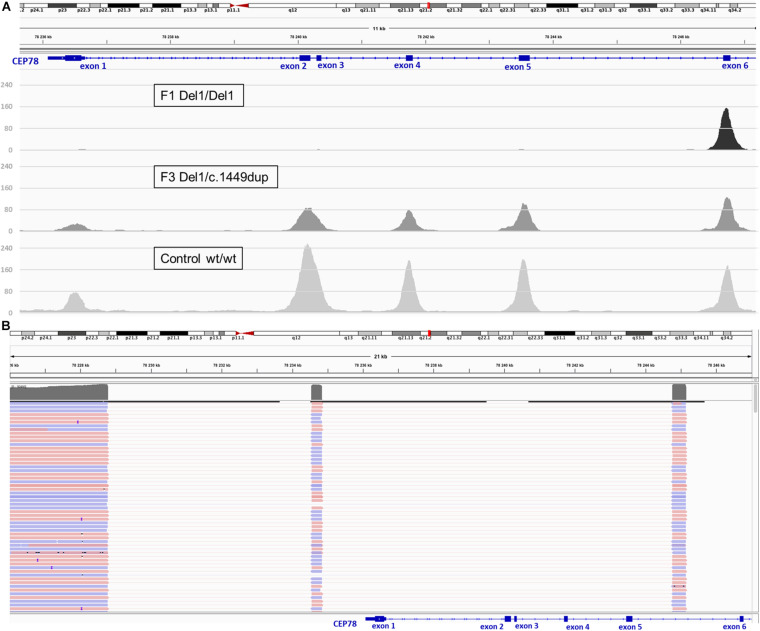
Deletion coverage plots and IGV view of the complex SV detected *via* targeted LRS in F3, II:1. **(A)** ExomeDepth coverage plots for homozygous deletion (F1, II:1, top) and heterozygous deletion (F3, II:1, middle) compared to control (bottom). Coverage suggested for F3, II:1 the same deletion described for F1, II:1 but in heterozygous state. **(B)** Delineation of the SV *via* targeted LRS of the junction product obtained in F3, II:1 identified the same complex deletion–inversion–deletion found previously in F1, II:1.

For F3, II:1 CNV analysis on WES data using ExomeDepth revealed a heterozygous deletion overlapping *CEP78* with the same coordinates of the deletion in F1, II:1 (Figure 3) in combination with c.1449dup [p.(Arg484Thrfs^∗^4)]. The latter is a novel 1-bp duplication in exon 12, creating a frameshift starting at codon Arg484. The new reading frame ends in a stop codon at position 4 (Supplementary Table 4). Segregation analysis confirmed the presence of c.1449dup on the maternal allele, while the deletion has paternal origin (Supplementary Figure 6). A junction product could be obtained *via* long-range PCR using the same primers used for F1, II:1. Targeted LRS on the long-range PCR amplicon allowed a final delineation, identifying once more the same complex deletion–inversion–deletion as found in F1, II:1 (Figure 3). Expression analysis of *CEP78* mRNA on available lymphocytes (F3 I:1, I:2, and II:1) and three controls showed extremely reduced *CEP78* expression due to the variants in the index case (Supplementary Figure 2).

### Haplotype Reconstruction for Recurrent Deletion–Inversion–Deletion of *CEP78* Identified in F1 and F3

The same deletion–inversion–deletion, originally described by Sanchis-Juan et al. (2018), has been identified here in F1 (homozygous) and F3 (heterozygous, with paternal inheritance); therefore, haplotype reconstruction using genotyping of 18 SNPs was performed in the proband of F1 (II:1) and in the proband and parents of F3 (II:1, I:1, and I:2). In addition, the haplotype of the homozygous *CEP78* SV reported by Sanchis-Juan et al. (2018), was reconstructed here on the basis of available WGS data. A common shared haplotype of at least 1.9 Mb was identified (Supplementary Table 5).

### Phenotypic Expression of *CEP78* Disease Caused by SVs and SNVs

**FIGURE 4:**
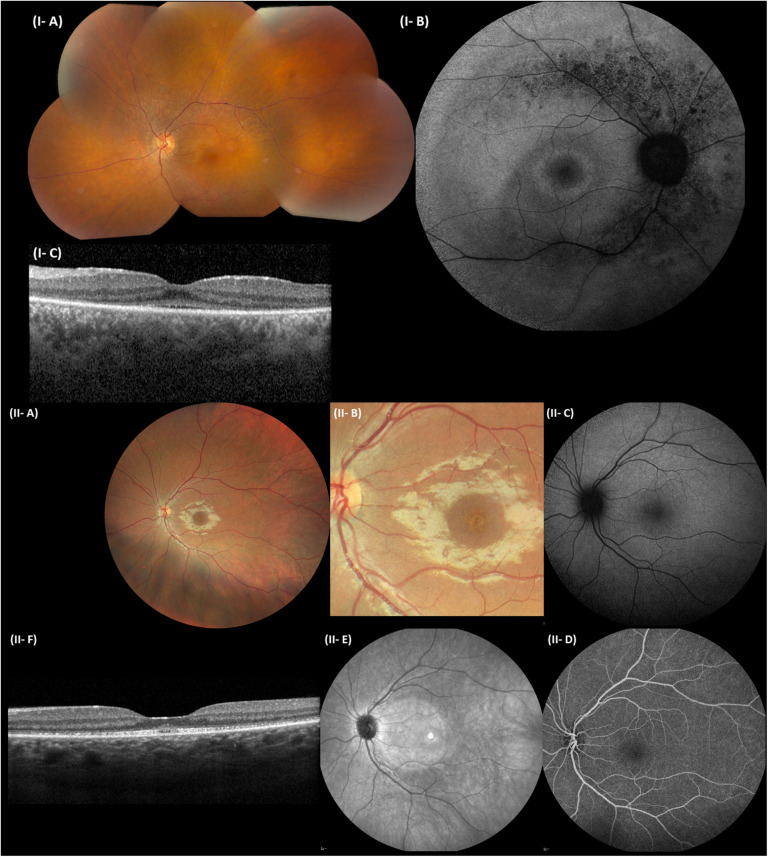
Representative ophthalmological pictures of individuals from F1 and F3 carrying *CEP78* structural variants. **(I)** F1, II:1. **(I-A)** Color fundus photograph of LE with normal optic disk, minimal narrowing of retinal arterioles, RPE mottling around the temporal vascular arcades, no bone spiculae. **(I-B)** BAF of the RE with hyperautofluorescent ring surrounding the fovea and granular hypo-autofluorescence around the temporal vascular arcades. **(I-C)** Spectral-domain OCT of the LE depicting loss of perifoveal photoreceptor layer and better foveal quality with loss, however, of integrity of photoreceptor outer segments. **(II)** F3, II:1. **(II-A)** Eye fundus of the left eye with perifoveal fine, granular, yellowish material upon close inspection. Normal aspect of optic disk, peripheral retina, and retinal vessels. **(II-B)** Close-up of the macular area, demonstrating the fine, granular, yellowish material encircling the fovea. **(II-C)** BAF of the LE without obvious abnormalities. **(II-D)** Fluorescein angiography of the LE with normal fluorescence. **(II-E)** Near-infrared imaging of the LE (hyperintense circular area is an artifact). **(II-F)** Spectral-domain OCT of the LE, revealing a mottled aspect of the ellipsoid zone and subfoveal collection of fluffy material. Enlarged and flat foveal depression. BAF, blue-light autofluorescence imaging; OCT, spectral-domain optical coherence tomography; RE, right eye; LE, left eye; RPE, retinal pigment epithelium.

In F4, II:1 WES filtering revealed a novel homozygous splice variant c.1208+2T>A [p.(?)] (NM_001098802.2), located in the donor splice site of intron 9 and predicted to cause skipping of exon 9. The variant involves the same intron as the one affected by c.1209-2A>C in F2, II:1 and is not reported in gnomAD. Segregation analysis in two of the obligate carrier children confirmed they are heterozygous carriers. Population frequencies, *in silico* predictions, and variant classification according to ACMG and ACGS criteria are listed in Supplementary Table 4 and Supplementary Figure 4. All patients included received an initial (F1, F2, and F3) or *post hoc*, genotype-driven (F4) clinical diagnosis compatible with CRDHL. In agreement with previous studies, the IRD phenotype displays a more pronounced cone dysfunction at the onset. The age at onset of the IRD ranged from the second to third decade and, of the hearing loss, from congenital to the first decade of life. More detailed clinical features are provided in Figure 4, Table 1, and Supplementary Clinical Data 1, 2. Affected individuals from F1 to F3 are females, whereas the affected individual from F4 is a male, without signs of subfertility/infertility. Apart from CRDHL, no additional clinical features were reported except for a balance disorder in F1. Overall, no genotype–phenotype correlation could be observed for the IRD phenotype in probands with biallelic *CEP78* SVs, SNVs, or a combination of both.

**TABLE 1:** Overview of clinical findings in *CEP78*-associated CRDHL.

	Age/sex	Origin	Age at onset	BCVA (RE/LE)	Goldmann visual fields	Fundus imaging	BAF	OCT	ERG	Hearing impairment	Other findings reported
F1 II:1	45 y/F	Belgian	22 y	0.1/0.1	Small central island of less than 5°, surrounded by large absolute (mid)peripheral scotoma (100° horizontal, 70° vertical) and normal peripheral limits	Normal optic disk, normal veins, narrowing of the arteries, perimacular grayish mottling around the vascular arcades and midperiphery, absence of bone spiculae	Mottled hypofluo rescence around the large vascular arcades, perifoveal hyperfluo rescent ring	Loss of outer retinal layers with sparing of subfoveal region with foveal swelling and blending	Maximum tendency toward electronegativity, scotopic function flat, cone function: both 30-Hz flicker and single flash present but delayed and reduced	Present since the age of 12 y, hearing aids	No fertility problems, balance disorder
F2 II:1	52 y/F	Danish	30 y	0.25/0.2 (49 y)	Paracentral scotomas, normal outer borders (49 y)	Normal optic disk, attenuated vessels, absence of hyperpigme ntations (49 y)	Mottled appearance midperiphery (49 y)	Attenuation of outer retinal layers (49 y)	Reduced cone and rod responses (39 y)	Congenital progressive hearing loss, hearing aids	None
F3 II:1	25 y/F	Belgian	24 y	0.3/0.2	Pericentral concentric scotoma	Discrete macular mottling	Unremarkable	Wide foveal depression	Lowered cone and rod responses, but worse for cones	High frequencies	Subtle mottling in central macula on infrared
F4 II:1	57 y/M	Turkish	NA	0.08/0.08	Small central island less than 5°, currently stable	Pale optic disk, narrow blood vessels, grayish discoloration, some bone spiculae in midperiphery	Mottled hypoautoflu orescence around the large vascular arcades and nasally, perifoveal hyperflu orescent ring	Attenuation of outer retina	Extinguished responses of cones and rods	Present since childhood, not progressive	None

*BAF, blue-light autofluorescence imaging; BCVA, best-corrected visual acuity; CF, counting fingers; ERG, full-field flash electroretinography; F, female; HM, hand movements; M, male; NA, not available; OCT, spectral-domain optical coherence tomography; RE, right eye; LE, left eye.*

### *CEP78* Expression in Human and Mouse Tissues Affected in CRDHL

**FIGURE 5:**
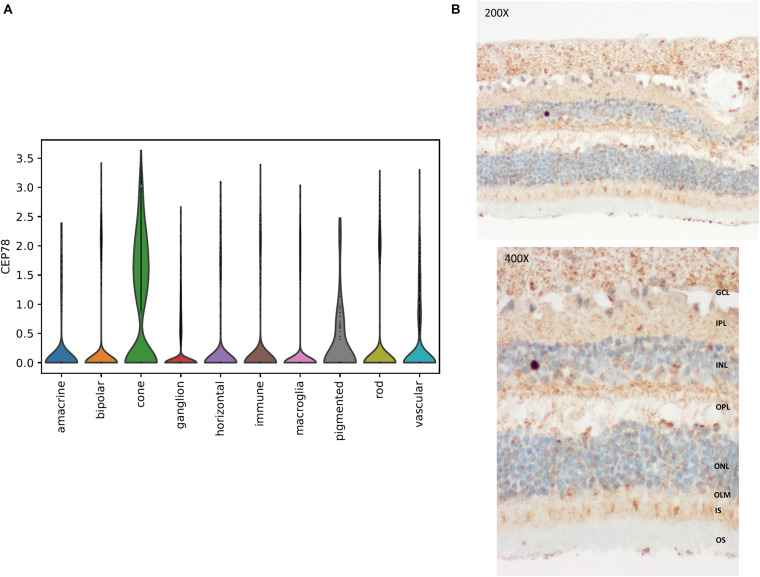
Single-cell transcriptional data in human retina and immunohistochemistry of CEP78 in adult human retina. **(A)** Violin plot for visualization of scaled and corrected expression of *CEP78* in 19,768 human neural retinal cells. Cells belonging to the cone cell population exhibit the highest expression. **(B)** Immunostaining of the CEP78 protein is visible in light brown. Light microscopy at 200 × (top) and 400 × (bottom). Predominant cytoplasmic expression of CEP78 can be seen in both cone and rod photoreceptors with strong staining at the base of the inner segments. Antibody: LN2004459 (1:100, rabbit, LabNed). GCL, ganglion cell layer; IPL, inner plexiform layer; INL, inner nuclear layer; OPL, outer plexiform layer; ONL, outer nuclear layer; OLM, outer limiting membrane; IS, inner segments; OS, outer segments.

In line with the main systems affected in CRDHL, i.e., neural retina and the inner ear, we examined *CEP78* expression in sc transcriptional datasets of human neural retina together with mouse and human cochlear or inner ear cells. This showed that *CEP78* is predominantly expressed in cone photoreceptor and in hair cell clusters, respectively (Figure 5 and Supplementary Figures 7, 8). Next, the expression patterns of *CEP78* and other ciliary genes listed in the top 10 of the HGC (Itan et al., 2013), *SCLT1*, *MKS1*, *CEP57*, *CEP76*, *CEP135*, *CEP152*, *CEP63*, *CEP164*, *OFD1*, and *CEP250*, in human adult bulk retinal transcriptional datasets were assessed for coordinated correlation, reasoning that this could offer insight into possible regulatory interactions. Interestingly, a matrix correlation plot (Supplementary Figure 9) showed *CEP78* expression to be correlated with *SCLT1* (ρ = 0.63, *p* < 0.001) and to be anticorrelated with *CEP250* (ρ = −0.53, *p* = 0.0007), both of them already associated with IRD (Khateb et al., 2014; Tonda et al., 2016; Kubota et al., 2018). Immunohistochemistry analysis of human retina shows predominant cytoplasmic expression of CEP78 in both cones and rods, with strong staining at the base of the inner segments, concordant with previous findings (Figure 5; Namburi et al., 2016; Nikopoulos et al., 2016).

## Discussion

In this study, we focused on *CEP78*-associated IRD, of which several studies have linked *CEP78* variants with a presumed loss-of-function effect to CRDHL. Interestingly, a homozygous complex SV affecting *CEP78*, i.e., a deletion–inversion–deletion, was recently described in a case of CRDHL (Sanchis-Juan et al., 2018). We assessed the role of SVs in CRDHL cases and found two distinct SVs affecting the *CEP78* region, emphasizing their role in *CEP78* disease. We showed the efficacy of CNV analysis of WES data to identify subtle CNVs; more specifically, we identified a deletion spanning exons 1–5 of *CEP78*, in both homozygous and heterozygous states, leading to loss of function. Furthermore, we showed the power of combined sWGS, qPCR, and targeted or whole-genome LRS to delineate and characterize SVs at the nucleotide level. This approach revealed a complex 15-kb deletion–inversion–deletion, which proved to be the same as the previously reported *CEP78* SV that was found and characterized by combined molecular karyotyping and Sanger sequencing. As this deletion was identified in the two families of Belgian origin and in a previously published case of a British patient, haplotype reconstruction was performed and suggested a founder effect. Apart from this, a distinct larger SV, being a 235-kb heterozygous deletion, was identified using molecular karyotyping and characterized using whole-genome LRS. This deletion encompasses the *CEP78* and *PSAT1* (MIM #610936) genes, the latter of which has so far only been implicated in recessive phosphoserine aminotransferase deficiency (MIM # 610992) and Neu-Laxova syndrome 2 (MIM # 616038) (Hart et al., 2007; Acuna-Hidalgo et al., 2014). Overall, for all cases with *CEP78* SVs identified by us, loss of *CEP78* expression was confirmed on patient-derived material. The latter is in line with the molecular effects of the previously reported *CEP78* sequence variants.

Sanchis-Juan et al. (2018) reported that the sequences surrounding all the breakpoints of the described SV in *CEP78* present high similarity to long interspersed nuclear elements (LINEs). It was hypothesized that repetitive elements enable replication-based SV formation, providing the necessary microhomology islands or increasing the vulnerability of the region to the formation of secondary DNA structures, which can lead to replication fork collapse (Sanchis-Juan et al., 2018). We performed a similar extensive *in silico* analysis of all breakpoints and junctions of the SVs identified in this study (Supplementary Figures 5, 10 and Supplementary Tables 6, 7). Interestingly, all breakpoint junctions, including the 235-kb *CEP78*-*PSAT1* deletion, showed microhomology, supporting repetitive element-mediated replication-based SV formation underlying *CEP78* SVs/complex SVs. Our findings underscore that SVs contribute to the genetic diversity of the human genome and are of high relevance for the molecular pathogenesis of rare diseases. Very recently, SVs were mapped and characterized in 17,795 deeply sequenced human genomes (Abel et al., 2020). It is estimated that SVs account for 17.2% of rare alleles genome-wide and that approximately 90% of such SVs are non-coding deletions. The number of complex SVs as a cause of genetic diseases is emerging. This is illustrated by *de novo* interspersed repeat insertions found in 124 cases with a genetic disease, 76 of which are caused by *Alu* short interspersed elements (SINEs), and 30 can be attributed to LINE-1 insertions (Payer et al., 2017). Two remarkable examples of complex SVs as a cause of IRD are SVA retrotransposon insertions in *MFSD8*- and *BBS1*-associated disease, of which the first SV served as a target for antisense oligonucleotide treatment, and the second appears to be a frequent cause of Bardet–Biedl syndrome (Kim et al., 2019; Delvallée et al., 2020). Another recent example is the identification of 33 pathogenic SVs in a cohort of 722 patients with autosomal dominant retinitis pigmentosa (adRP). Indeed, eight distinct complex non-coding SVs were found as the underlying mechanism of RP17-linked adRP in 22 affected families with >300 affected individuals, clearly emphasizing the importance of the non-coding genome as target for SVs in IRD (de Bruijn et al., 2020).

Given a high contribution of CNVs to ∼7%–10% of the pathogenic alleles in IRD, the fact that the majority of the SVs in our genome are non-coding and the emerging number of non-coding or complex SVs causing IRD (Abel et al., 2020; Zampaglione et al., 2020), it can be expected that SVs represent an important part of missing heritability of IRD. Hence, there is a need to assess the impact and frequency of SVs in IRD cohorts more systematically. Here, we showed how CNV calling on WES data, sWGS, and targeted or whole-genome LRS led to a confirmed molecular diagnosis in *CEP78*-associated IRD cases. CNV assessment using NGS-based algorithms has been already described as a reliable method to enhance the diagnostic rate of IRD (Khateb et al., 2016; Ellingford et al., 2018). Furthermore, this study supports the superiority of LRS for fast characterization of complex SVs. Massively parallel sequencing is currently dominated by second-generation sequencing technology, mainly relying on SRS, having its limitations for the identification of cryptic SVs, sequencing repetitive regions, phasing of alleles, and distinguishing highly homologous genomic regions, mainly due to its short-read lengths. Third-generation or LRS technologies offer improvements in the characterization of genetic variation and regions that are difficult to assess with the current SRS approaches. The main advantage of LRS compared to SRS technologies comes from the use of long reads (>10 kb on average), originating from single-DNA molecules. Apart from this, the sequencing occurs in real time without the need of PCR amplification, therefore being mostly free from PCR-related bias. The power of LRS approaches to overcome specific limitations of second generation–based analyses will further become clear when LRS will be implemented in routine genetic testing workflows.

Apart from the *CEP78* SVs, the two novel splice variants found here expand the spectrum of *CEP78* (likely) pathogenic SNVs: a novel canonical acceptor and donor splice variant of intron 9, respectively, c.1209-2A>C (p.?), predicted to lead to an exon 10 skip, and c.1208 + 2T > A (p.?), predicted to cause an exon 9 skip. Interestingly, skipping of the 46-bp exon 10 was previously reported for c.1254 + 5G>A (*CEP78*, NM_032171) (Fu et al., 2016). When comparing the phenotypes found in CRDHL cases with SNV and SVs, no apparent genotype–phenotype correlation could be demonstrated that would allow discriminating between the two classes of variants, emphasizing the need for a systematic SV assessment in the genetic workup of CRDHL cases, in which a *CEP78* genotype is suspected or in “atypical” Usher syndrome cases.

Using data mining of bulk or sc transcriptional datasets from human retina, mouse cochlea, and human inner ear, we showed expression in cone photoreceptors and in hair cell clusters. These expression domains are in agreement with the main systems affected in CRDHL, i.e., neural retina and the inner ear. Apart from this, a correlation study of *CEP78* expression strengthened the importance of CEP78 in the ciliary machinery.

To conclude, this study supports that the *CEP78* locus is prone to microhomology-mediated, replication-based SV formation and that (complex) SV analysis should be included in molecular genetic testing of CRDHL or “atypical” Usher syndrome. Finally, it demonstrates the power of WES-based CNV assessment, sWGS, and whole or targeted LRS in identifying and characterizing suspected complex SVs in patients with *CEP78*-associated IRD. Systematic SV assessment in IRD will certainly close a diagnostic gap and will contribute to precision medicine in IRD.

## Data Availability Statement

The variants presented in this study can be found in online repositories. The name of the repository and accession numbers can be found below: Leiden Open Variation Database (LOVD), https://databases.lovd.nl/shared/individuals/00332412; https://databases.lovd.nl/shared/individuals/00332413; and https://databases.lovd.nl/shared/individuals/00332414.

## Ethics Statement

The studies involving human participants were reviewed and approved by Ethics Committee Ghent University Hospital, Ghent University Hospital, Ghent. Written informed consent to participate in this study was provided by the participants’ legal guardian/next of kin. Written informed consent was obtained from the individual(s), and minor(s)’ legal guardian/next of kin, for the publication of any potentially identifiable images or data included in this article.

## Author Contributions

GAs and EDB designed the study and wrote the overall manuscript. GAs, NR, MDB, MVH, DC, JVD, TVL, TDP, AR, and EDB performed the research. JDZ, MVL, JJ, MBe, and LT clinically evaluated and described the families included in the study. GAs, NR, MDB, MBa, MVH, GAr, DC, JVD, TR, TDP, PDR, WDC, BM, AR, MS, and EDB analyzed and described the data. All authors reviewed the manuscript.

## Conflict of Interest

The authors declare that the research was conducted in the absence of any commercial or financial relationships that could be construed as a potential conflict of interest.

## Footnotes

1 https://sph.uth.edu/retnet

2 https://www.biorxiv.org/content/10.1101/201178v3

3 http://www.hgvs.org
