# Supplementary material for: Long-Read Sequencing to Unravel Complex Structural Variants of CEP78 Leading to Cone-Rod Dystrophy and Hearing Loss
Source: Front Cell Dev Biol. 2021 Apr 21;9:664317. doi: 10.3389/fcell.2021.664317 (PMC8097100; doi:10.3389/fcell.2021.664317)
Supplement: Supplementary file 1 [file Data_Sheet_1.pdf]

SUPPLEMENTARY DATA

SUPPLEMENTARY CLINICAL DATA

Supplementary Clinical Data 1. Representative ophthalmological pictures of F2, II:1 carrying the *CEP78-PSAT1* deletion combined with *CEP78* c.1209-2A>C. A. Fundus image of the right eye at age 49 showing generalized retinal dystrophy with attenuated retinal vessels. **B.** Fundus autofluorescence of the right eye at age 49 showing a midperipheral mottled appearance. **C.** OCT image of the left eye at age 49 showing attenuation of outer retinal layers.

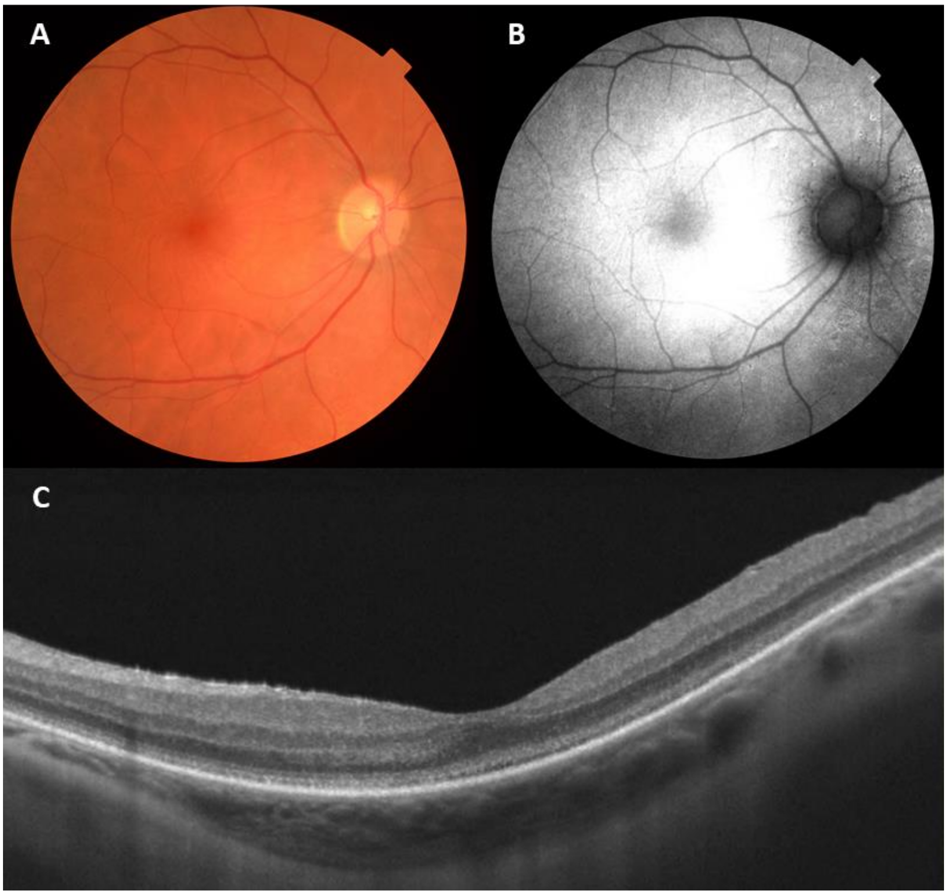

Supplementary Clinical Data 2. Representative ophthalmological imaging of F4, II:1 homozygous for c.1208+2T>A (p.?) in *CEP78* and his extended family pedigree. A. Fundus image of the left eye at age 57 showing narrow blood vessels and greyish discoloration. **B.** OCT scan showing attenuation of the outer retina. **C.** Fundus autofluorescence of the left eye showing mottled hypoautofluorescence around the large vascular arcades and a perifoveal hyperfluorescent ring. **D.** ERG with extinguished responses of cones and rods. **E.** Extended pedigree of F4.

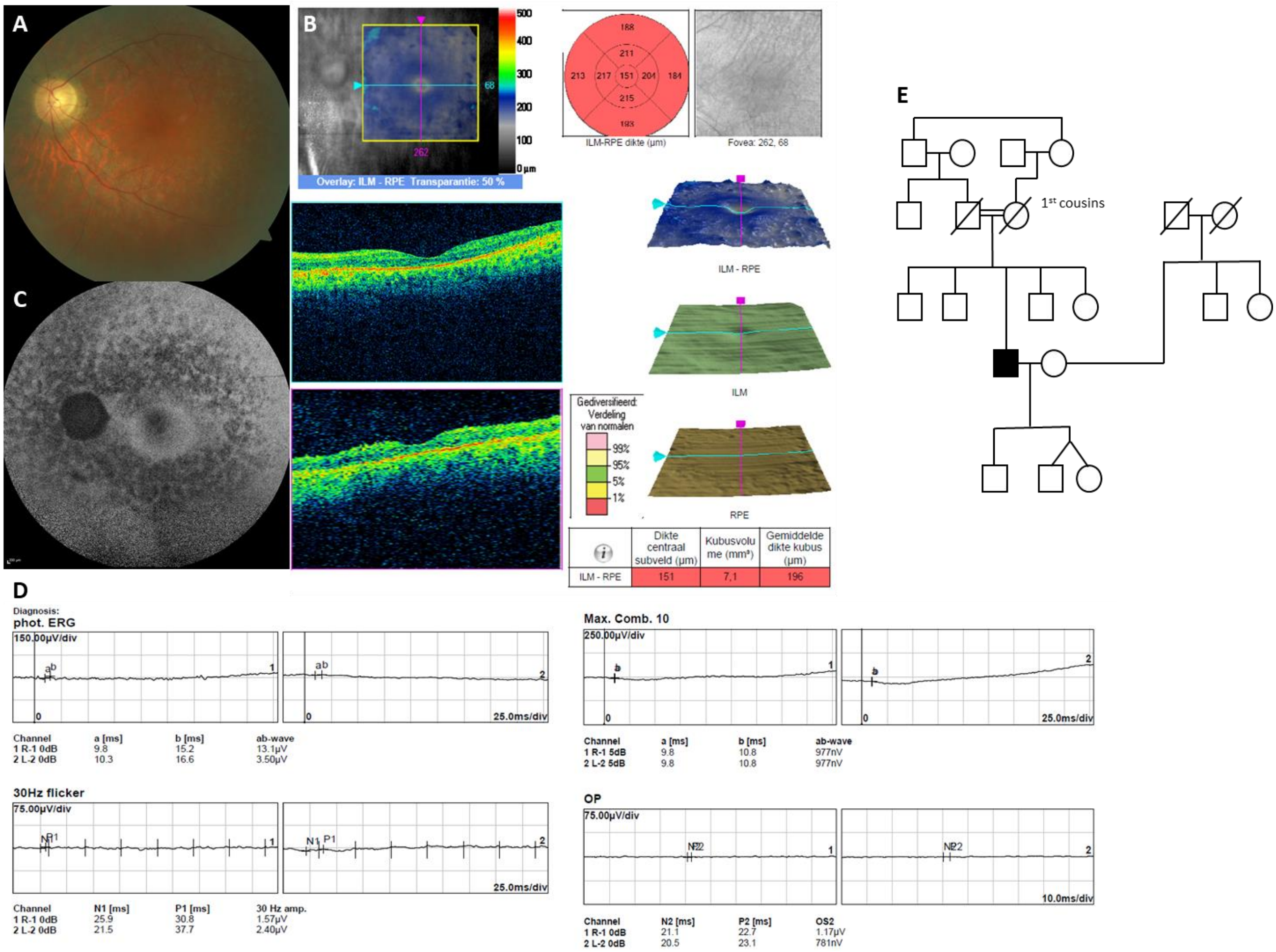

SUPPLEMENTARY FIGURES

Supplementary Figure 1. Junction deletion product from F1. Long-range PCR confirmed the presence of a junction product of the expected size (~12 kb) (left lane). Primers used: Start3-fw (TTTGCGCTTTCTGTACAACC) and Intron5-2-rv (GACACCACAAGACACACAGAAA). Ladder: 1 kb Plus DNA (Thermo Fisher) on 1% agarose gel (right lane).

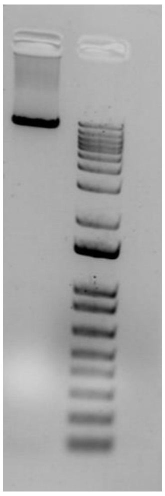

Supplementary Figure 2. *CEP78* mRNA expression analysis on control and patient-derived materials (F1-F3). A. RNA expression profile in the affected individual F1, II:1 and in three controls. **B**. RNA expression profile in the affected individual F2, II:1 and in two controls. **C**. RNA expression profile in the affected individual F3, II:1, her parents (F3, I:1 and I:2) and three controls. Quantitative RT-PCR (qRT-PCR) was performed on cDNA synthesized from total RNA from short-term cultured lymphocytes (F1 and F3) or fibroblasts (F2). Data were analyzed with qbase+ and normalized to the *YWHAZ* and *HMBS* or *SDHA* genes. In F1, II:1 and F2, II:1 loss of *CEP78* expression is observed, compared to controls. In F3, II:1 *CEP78* expression is strongly reduced compare to her parents (F3, I:1 and I:2) and controls.

A

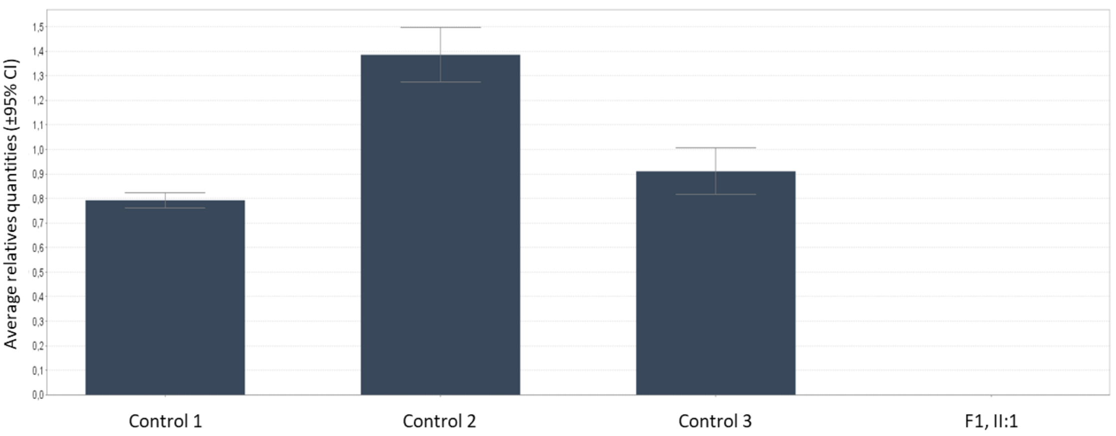

B

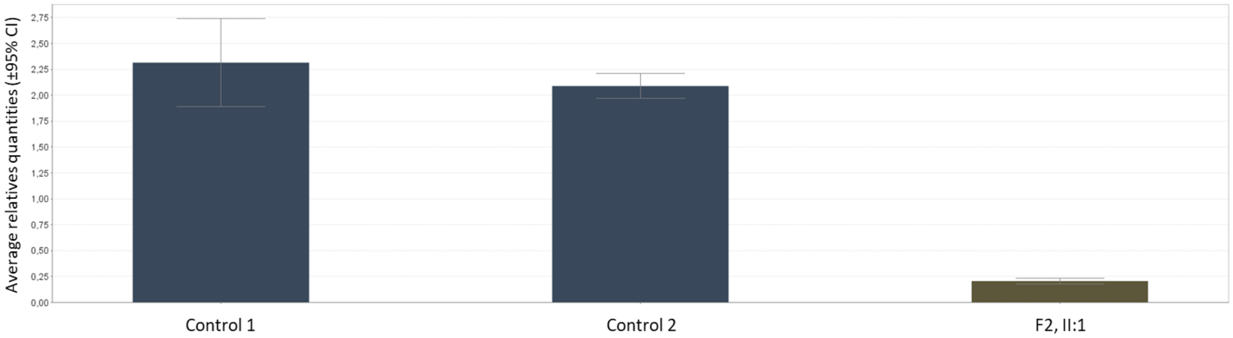

C

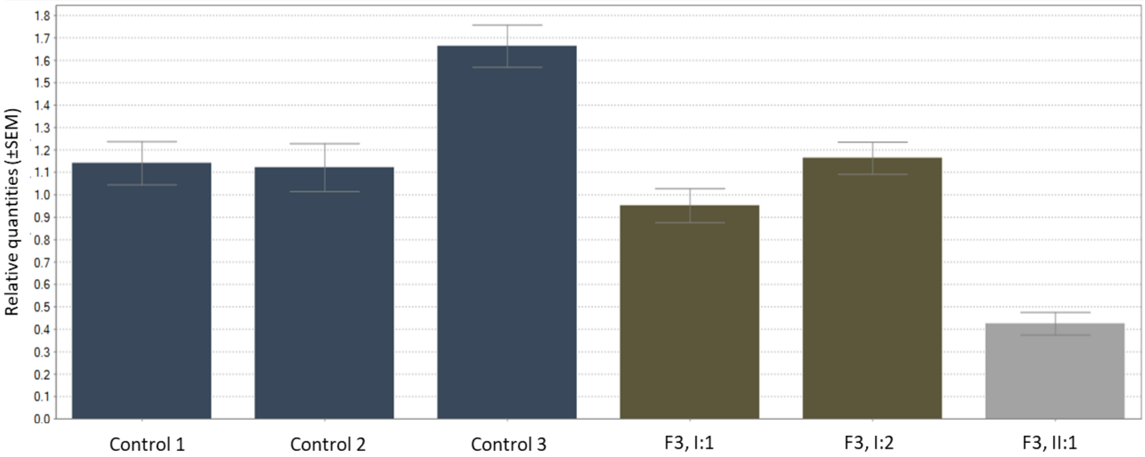

Supplementary Figure 3. Segregation analysis in F2 and RT-PCR in F2, II:1. A. Segregation analysis suggested a heterozygous genomic deletion in the *CEP78* region for F2, II:1, in compound heterozygosity with the splice variant c.1209-2A>C. Primers used: *CEP78*-ex10-F: CCAGGCCCTTATTTGGAAGT and *CEP78*-ex10-R: AGGCCTCCATTGTGTGACAT. **B.** RT-PCR is showing skipping of exon 10 due to the splicing variant. Primers used: *CEP78*-c.444F-RT-PCR (ACCTGTCTCTTGCAAATTGTCC) and *CEP78*-c.1300R-RT-PCR (AGGATGAAGGACTCTCTACTGTC). Ran on 2% agarose gel. **C.** Direct sequencing of the amplified *CEP78* cDNA from the proband confirmed exon 10 skipping.

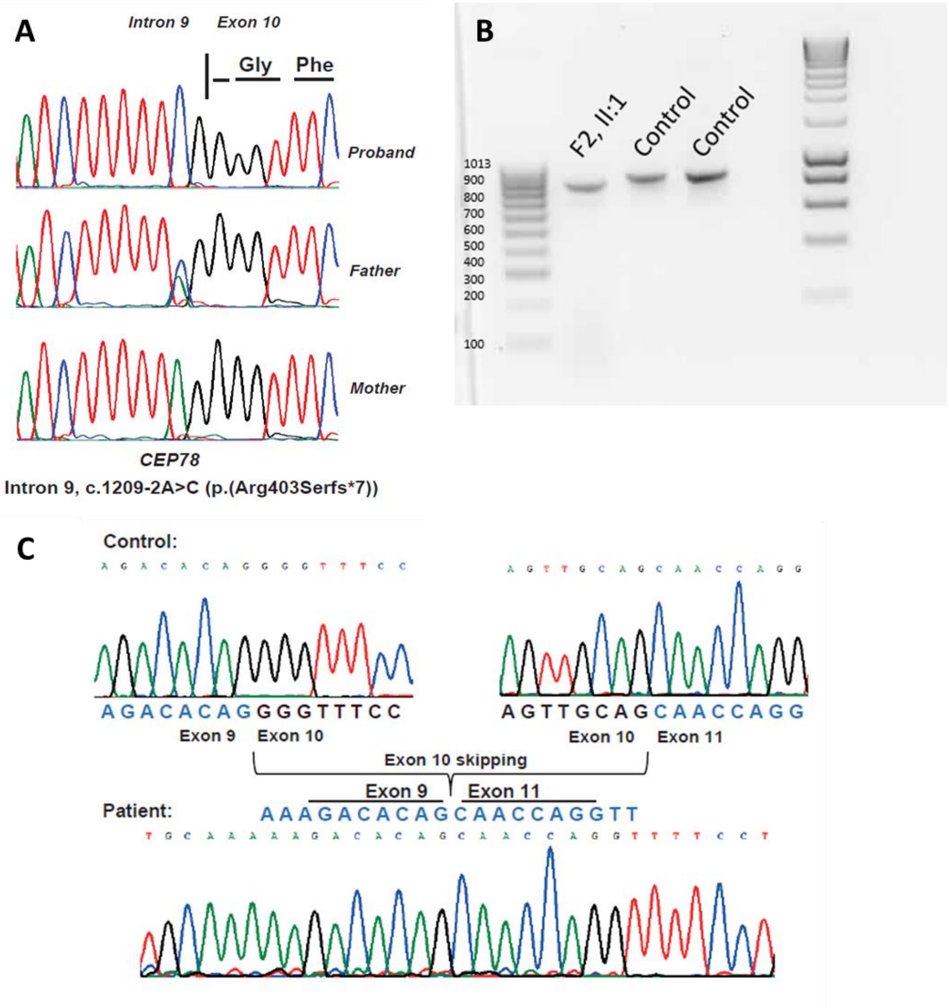

Supplementary Figure 4. Alamut Visual splicing predictions. A. Splicing prediction for *CEP78* c.1209-2A>C (F2, II:1), predicting complete loss of the canonical acceptor splice site of intron 9. **B**. Splicing predictions for *CEP78* c.1208+2T>A (F4, II:1), predicting complete loss of the canonical donor splice site of intron 9. Predictions from Alamut Visual v.2.11.0.

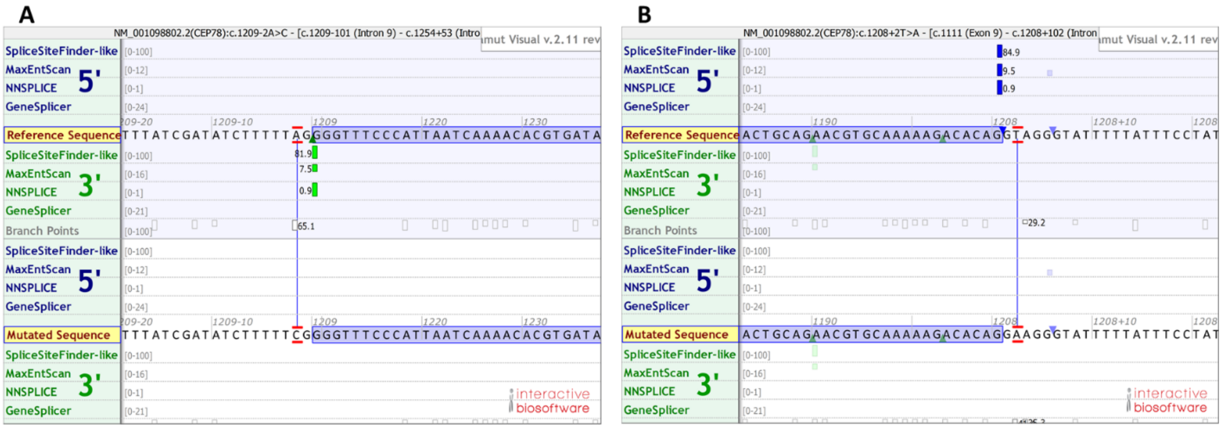

Supplementary Figure 5. Breakpoint evaluation of the deletion spanning *CEP78-PSAT1* in F2, II:1. Comparison of the sequences at the breakpoint junctions shows microhomology, supporting microhomology mediated break-induced replication as the underlying mechanism. In blue-grey the junction point is highlighted, in bold the breakpoint as suggested by LRS.

TTTGTCATTTACTACATGCTTTCCCTAGGTGTTAGCC**T**TAAATTGTGGCCAAGAATCATTCTACTTT
TTTGTCATTTACTACATGCTTTCCCTAGGTGTTAGCCTAGCCACTGCGTCTGGCTGAATGAAAAATC
TTGTCTTGGCCTCCCAAAGTGCTGGGATTGCAGGCAAGAGC**C**ACTGCGTCTGGCTGAATGAAAAATC

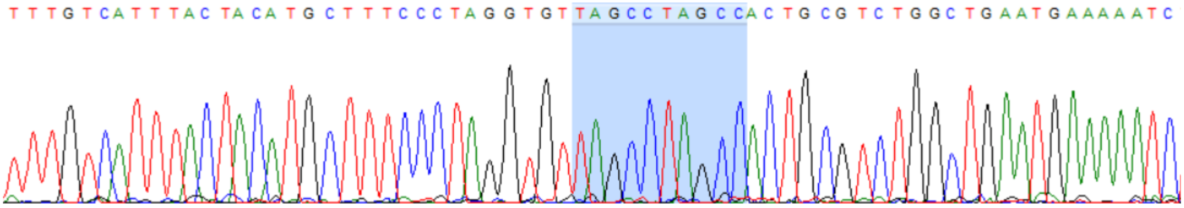

Supplementary Figure 6. Segregation analysis in F3. Segregation analysis has been performed combining long-range PCR (as performed for F1) and Sanger sequencing (*data not shown*). In the proband of F3 the deletion is paternal while the c.1449dup variant is maternal.

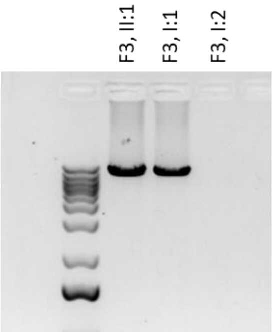

Supplementary Figure 7. *CEP78* transcriptional landscape. A. Median *CEP78* gene-level expression in transcripts per million (TPM) across human tissues (GTEx) and retina. **B.** Average *CEP78* retinal expression quantified at transcript level: CEP78-215 (ENST00000643273.2) is the most highly expressed isoform. **C.** Dot plot showing predominant expression of *CEP78* in cones (human): individual dots are colored and sized to visualize both the proportion of cells in each population expressing a given gene (dot diameter size) and the average expression level of that gene (dot color intensity) in the detected cells.

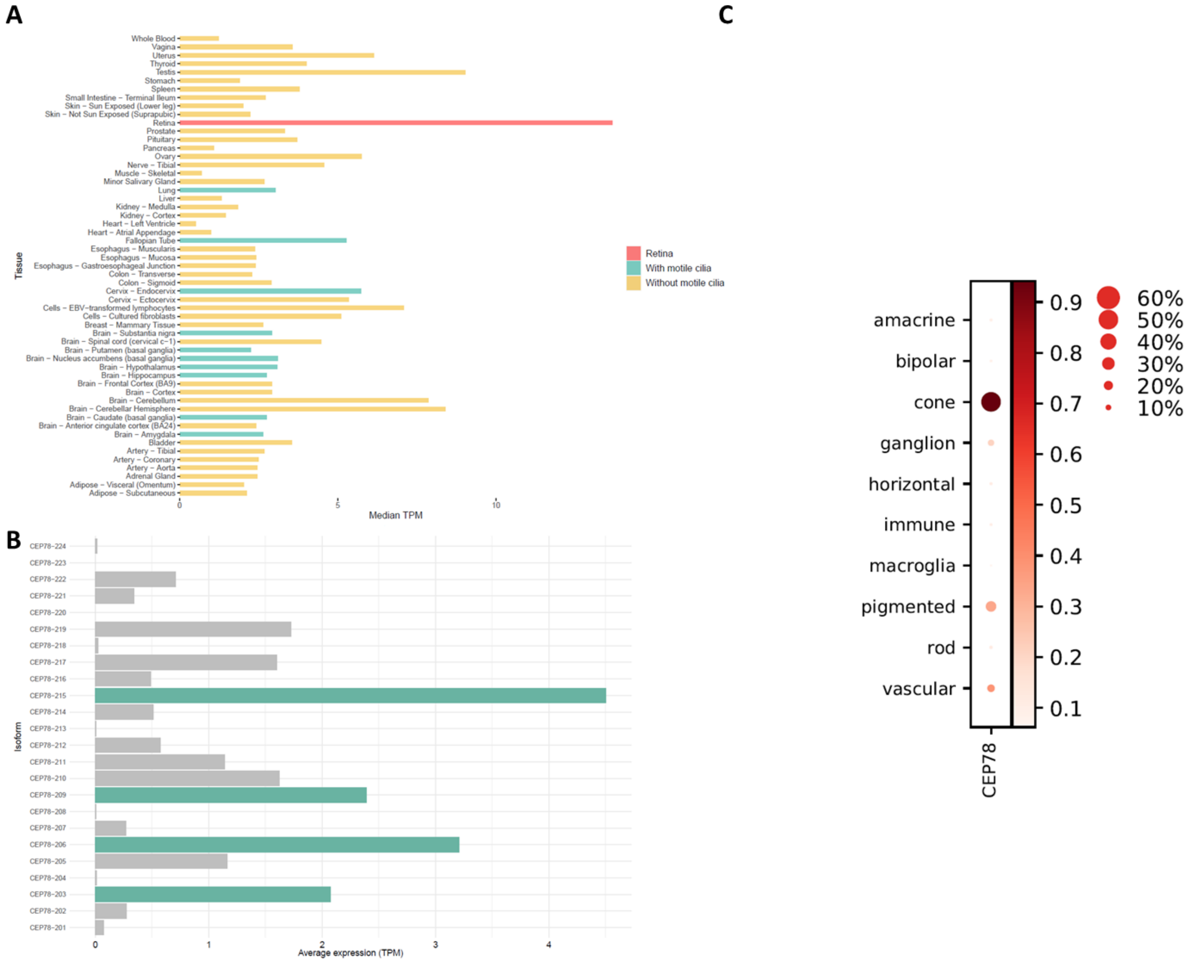

Supplementary Figure 8. ***CEP78*** **expression in human and murine transcriptional datasets A.** (top) UMAP plot of 11,332 P1 murine cochlear cells coloured based on the 21 transcriptionally distinct clusters (0-20) and visualization of *Cep78* expression; (bottom) visualization of feature expression on UMAP plot of marker genes for inner/outer hair cell population: *Slc7a14, Dnm3, Pvalb*. **B.** (top) Bulk *CEP78* expression in murine inner/outer hair cells and (bottom) human cochlea/components of the vestibular labyrinth (saccule, ampulla, utricle).

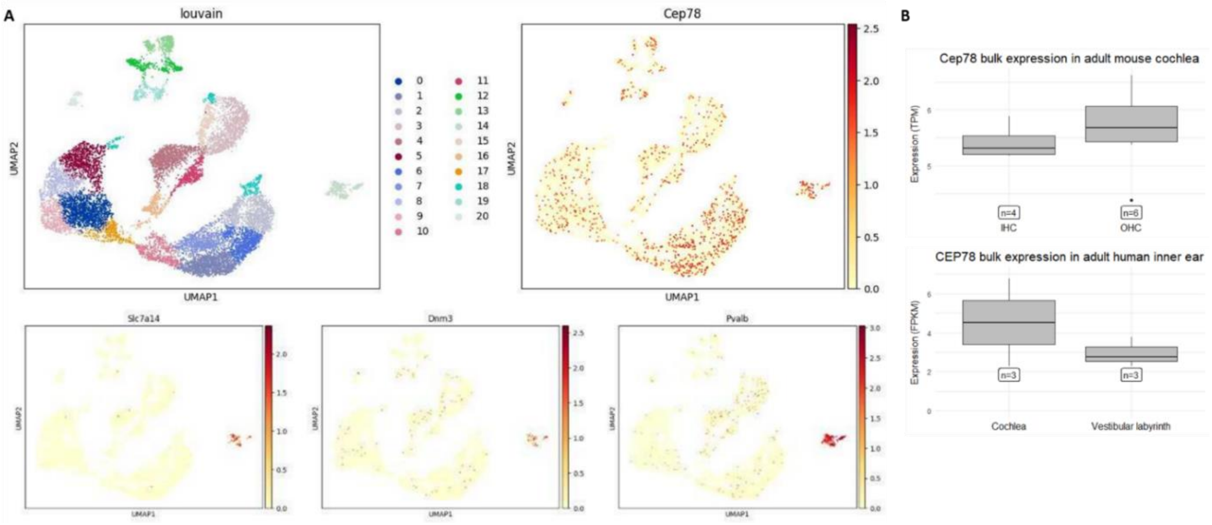

Supplementary Figure 9. Correlogram of ***CEP78*** **expression and selected ciliary genes in human retina.** The expression patterns of *CEP78*, *SCLT1*, *MKS1*, *CEP57*, *CEP76*, *CEP135*, *CEP152*, *CEP63*, *CEP164*, *OFD1*, *CEP250* in human adult bulk retinal transcriptional datasets were assessed for coordinated correlation, providing insight into potential regulatory interactions. As shown in the correlogram, *CEP78* expression was found to be anti-correlated with *CEP250* (rho=-0.53, p=0.0007) and to be correlated with *SCLT1* (rho=0.63, p<0.001) and other centrosomal genes.

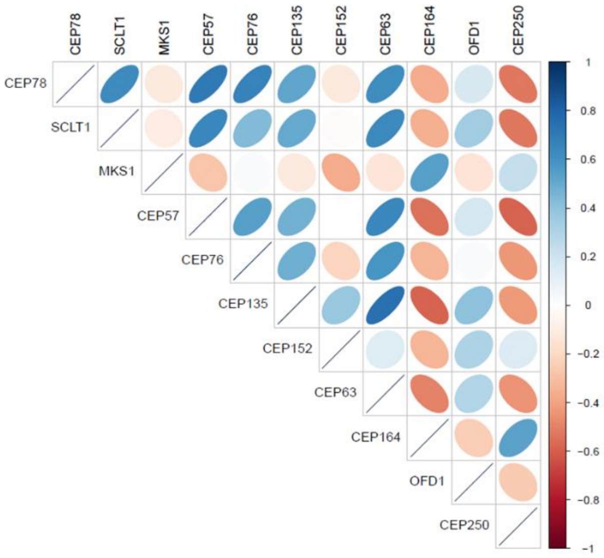

Supplementary Figure 10. Overview of identified *CEP78* SVs in this study. Top panel: Overview of the two *CEP78* SVs identified in this study: the 235-kb deletion found in F2, II:1 (chr9:78096930-78331887) and the recurrent complex SV found in F1 and F3. Deletions are depicted in red, the inversion in blue. Numbering of the breakpoints of the 235-kb deletion is shown here (1 and 2). Lower panel: Breakpoint numbering of the recurrent complex SV is shown here (orange box). A close-up is shown for the complex SV (red: deleted regions, blue: inverted region). Breakpoint and junction numbering used for an in-depth analysis of these regions (see Supplementary Table 6) are shown (BP: 1-2-3-4, J1 and J2). Of note, LINE repeats intersect all breakpoints of the complex SV. Abbreviations: D1: deletion 1; D2: deletion 2; I and INV: inversion; SINE: short interspersed nuclear element; LINE: long interspersed nuclear elements; LTR: long terminal repeat; DNA: deoxyribonucleic acid and RNA: ribonucleic acid.

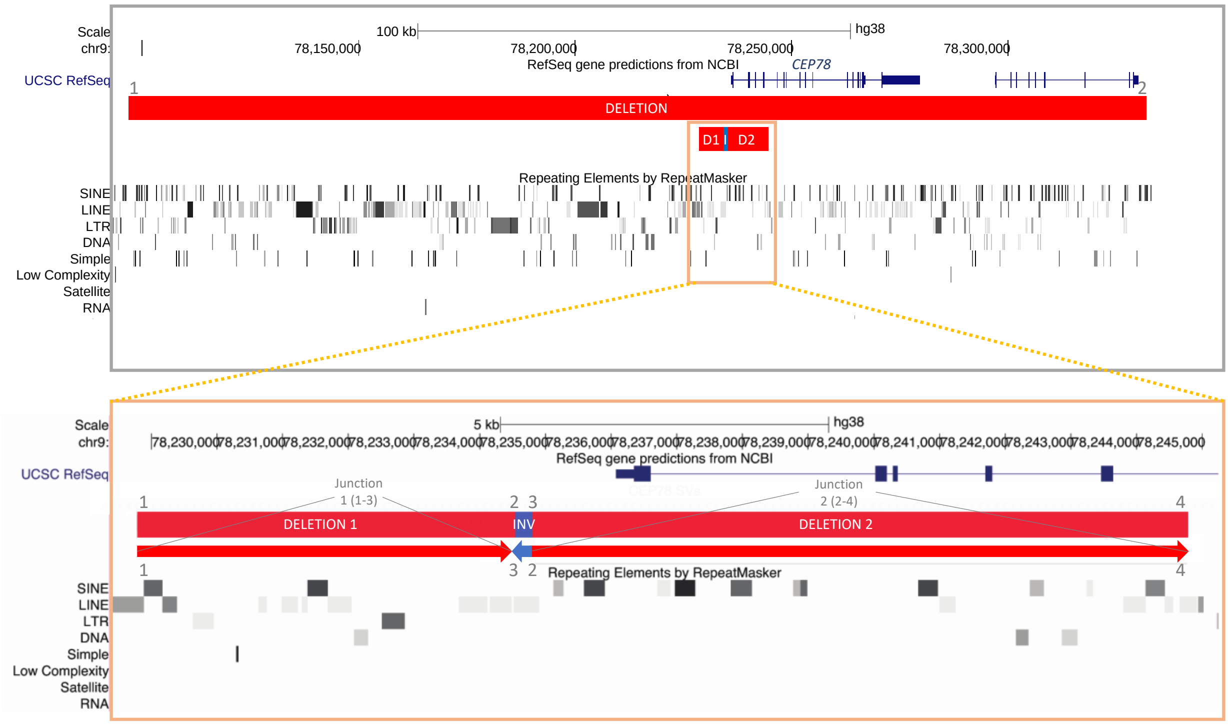

SUPPLEMENTARY TABLES

Supplementary Table 1. PCR primers for CNV analysis or expression analysis on patient-derived material and controls. *GNAQ* and *PSAT1* are the flanking genes of *CEP78*. Primers were designed with Primer3Plus (www.bioinformatics.nl/primer3plus).

Target	Forward primer (5'-3')	Reverse primer (5'-3')
**CNV screening**		
GNAQ	CCAGAGTCATTCTTCCAAAGTG	GCGGGAGGGTGTGTGT
CEP78 start1	GGAGTTAGCCAACATCATCG	TCCTGGCAAATTGAAGAGTG
CEP78 start3	TTTGCGCTTTCTGTACAACC	TGAATAATGGTGAGGGACGA
Exon 1	CTCCCACTACGAGTACCT	GATCTTGAGGGTGCTCAG
Exon 2	CCTGCGATAAGATACAAAGATG	CAGGTTCTTTAGCACACTTG
Exon 3	CCCTCATTAAAGGGATTGAATA	CACTTTCTAAACCTCCATCTC
Exon 4	AGTTATTTGTCAAGGTATAAAGAGC	ATCTTGGCCATGTGATCTG
Exon 5	GAAACCTGGGCTGAGAG	GGTCACCAATAAGTGTGTTG
Exon 6	TTTGCTAGAGGCCCTTGAAA	TGTAGCGACATACCAATGAGT
Exon 7	AGATCATTCTATGATGAAAGCAG	AAACATGATATACCTCTGATTTGG
Exon 8	TTTAGTACCAGTGGATAACTTCT	CCTTTGTGACCACTTCCT
Exon 9	CCTGTAAGTAGTGGCAGAAA	CACGGCAAGAAACCAGA
Exon 10	GCAAGTGTTATTACCTAGTGTT	GGTAACTACGTACCTGCAA
Exon 11	CCTGTGACTGTGACAGTAGAGA	TGTACCTGTAATGCTTCTTGTTCT
Exon 12	ACTGGAGGAGTGCCTAAA	TATTTACCTCACTGACTCGTTTA
Exon 13	AATTTCTCTTTGTCTGAAGCC	TCTCAATGCTGCCCAAA
Exon 14	GCTGGGATAGATCAGTCAG	TAGGGCATTCTGCTTCG
Exon 16	TTTCCTGTCCCAGTTTCTAC	GGTGAAGGAGAACACATTCTA
End *CEP78*	GACAAGCAGGGACATGAGATAC	TTTGCACTACATGTATGGGAGTC
*PSAT1*_1	GGGGGTTGAAACAGTAAACG	AGGAGCTCACATCCCCATT
*PSAT1*_2	GACTCGGCGCAGGAAC	GTGAGTCAGCCAAGGAGGA
CEP78 Intron5-1	GGGAGGGTAATCATGACACA	ATTCATGCCTGGAAGACTCC
CEP78 Intron 5-2	TGCAGCAAGAGTTTGTAAAGG	GACACCACAAGACACACAGAAA
**Expression analysis**		
CEP78 expression	GGCTTAAGACGTATCACACT	CACTGTTGCAGGTCAAGA
**Reference genes**		
HMBS expression	GGCAATGCGGCTGCAA	GGGTACCCACGCGAATCAC
YWHAZ expression	ACTTTTGGTACATTGTGGCTTCAA	CCGCCAGGACAAACCAGTAT
SDHA expression	TGGGAACAAGAGGGCATCTG	CCACCACTGCATCAAATTCATG
GPR15 CNVscreening	GGTCCCTGGTGGCCTTAATT	TTGCTGGTAATGGGCACACA
ZNF80 CNVscreening	CCCAGTGTGAATCTGCT	GAAGACCTACAAATGCAAGG

Supplementary Table 2. PCR primers for haplotype analysis. Primers were designed with Primer3Plus.

Target	Forward primer (5'-3')	Reverse primer (5'-3')
rs524314	GAGGGGAAGGACCAGACTCT	TGGCTTAGAACAGTTTTCATGAATG
rs1029037	CCACATTCCACAACATGGAG	TGAAAACATGAAATATACAAGAACAGA
rs9314838	TGCTTCAGTTGTAGGGGCAG	TTAGGGATGGCATCTCCTGG
rs631887	CAGTTGTTGGATTAGGCAGGC	TCATCAGGGTGAATACAGAAGTCA
rs10156442	GAAACCCCTGACCACAGAC	GGAGGAGATGAAAAGCAAGTT
rs1953019	CATTTTGTGCACATCCTCAAG	TGTTCTTTTTGATGCATTTTCTT
rs2026000	GCAGGAACTTCAGATTTTGGA	TCACGTCCCATGTTCAGTGT
rs4877499	CCTGGATCCATCAAACCTCA	GGGGATCTGGTGAGTGAGTG
rs2521904	TGCTTCCAAAGTTAGAACAGCAT	AACATTTTCTCAAAGAACAGTCCTA
rs7869495	TTAATGCTGCATGGGTCTTG	TGAAAGCAGGGAGCATCTG
rs11792810	GGTCAACTTAATGTAAACTAGCAAAG	GCTGAAGGTGTATGTGTGTATGTATTT
rs10780305	AGTTGTTGTGTGCTTGCAGC	CATTGATAATTTCCTCCTTATTCTGGT
rs946806	CAAGTTACAGTAGGCAAGCACG	CGGTACTGAACAGAGCAGGC
rs7039267	ACAGTGCAGGGATAGGTATAAAG	TGGCATGGTATTAACAGTCTAACA
rs7041989	GGAGGTTTAAGTTTTTAGCACCA	AGCTCACTGGGTCATGTGAA
rs2988072	TGCCCGGTTACATGGTAGAT	TCCTCACTTCCACACTGCTG
rs7032321	TCACCACACCTTTTCAGAAGC	TCCAACCCTCGATGAGTTATT
rs12335799	CTGTTATTTTCCGTGGGAGA	TGGGACTAGCTGTATGGCATC

Supplementary Table 3. sWGS output regarding the deletions overlapping the *CEP78* region. Information is derived from ViVar (https://vivar.cmgg.be/).

	**F1, II:1**<br>Homozygous deletion (9) (q21.2)<br>chr9: g.78230001-78245000<br>min 15 kb <--> 20 kb max	**F2, II:1**<br>Deletion (9) (q21.2)<br>chr9: g.78105001-78330000 (hg38)<br>min 225 kb <--> 240 kb max
Gene content	*CEP78*	*CEP78* and *PSAT1*

Supplementary Table 4. Classification of the *CEP78* variants following ACMG and ACGS guidelines with adaptations. Information is derived from Alamut Visual (v.2.11.0; NM_001098802.2) and dbNSFP. *In silico* high impact predictions are indicated in bold. Abbreviations: PM: pathogenic moderate; PP: pathogenic supporting; PS: pathogenic strong; PVS: pathogenic very strong. The strength of some criteria was altered under certain conditions as recommended in literature (Abou Tayoun et al., 2018; Biesecker, Harrison, & ClinGen Sequence Variant Interpretation Working Group, 2018; Ellard et al., 2019, 2020; Nykamp et al., 2017). If available, information from literature and databases is not included in the criteria to avoid double counting of existing classifications that are based on the same set of data (Biesecker et al., 2018).

	Variant details		
	**F2, II:1**	**F3, II:1**	**F4, II:1**
gDNA Chr9 (GRCh38)	g.78253230A>C	g.78262972dup	g.78252045T>A
cDNA	c.1209-2A>C	c.1449dup	c.1208+2T>A
Protein	p.?	p.(Arg484Thrfs*4)	p.?
Location	Acceptor splice site of intron 9	Duplication (1 bp) in exon 12	Donor splice site of intron 9
dbSNP	rs778035330		
gnomAD v3.1	ALL: 0.002628 % - NFE: 0.001470%		
CADD: scaled C-score	**25.6**		**23.7**
Mutation Taster	**1**		**1**
DANN	0.8316		**0.99**
FATHMM-MKL	**0.9198**		**0.968**
Eigen raw	**0.7215**		0.88
GERP++	5.01		5.25
RF	0.748		**0.946**
FitCons	0.2629		0.16
ADA	**0.9999**		**1**
MaxentScan	-0.564 (alt); 7.479 (ref)		1.28 (alt); 9.46 (ref)
Splicing (SpliceSiteFinder-like, MaxEntScan, NNSPLICE, GeneSplicer and Human Splicing Finder)	Predicted change at acceptor site 2 bps downstream: **-100.0%** MaxEnt: -100.0% NNSPLICE: -100.0% SSF: -100.0% **Skip of exon 10 is very likely**	No effects	Predicted change at donor site 2 bps upstream: **-100.0%** MaxEnt: -100.0% NNSPLICE: -100.0% SSF: -100.0% **Skip of exon 9 is very likely**
**Variant classification using ACMG-ACGS guidelines**			
Population data	PM2: Absent from controls or at extremely low frequency if recessive in gnomAD	PM2: Absent from controls or at extremely low frequency if recessive in gnomAD	PM2: Absent from controls or at extremely low frequency if recessive in gnomAD
Genotype and phenotype of the patient	PP4_PM: Patient' phenotype or family history is highly specific for a disease with a single genetic etiology - after discussion with the physician	PP4_PM: Patient' phenotype or family history is highly specific for a disease with a single genetic etiology - after discussion with the physician	PP4_PM: Patient' phenotype or family history is highly specific for a disease with a single genetic etiology - after discussion with the physician
Literature	ClinVar ID: 836906	/	/
Computational predictions	PVS1: null variant (nonsense, frame shift, canonical +/- 1 or 2 splice sites, initiation codon or multi-exon deletion) in a gene where 'loss of function' is a known disease mechanism	PVS1: null variant (nonsense, frame shift, canonical +/- 1 or 2 splice sites, initiation codon or multi-exon deletion) in a gene where 'loss of function' is a known disease mechanism	PVS1: null variant (nonsense, frame shift, canonical +/- 1 or 2 splice sites, initiation codon or multi-exon deletion) in a gene where 'loss of function' is a known disease mechanism
Functional data	PS3: Well-established in vitro or in vivo functional studies supportive of a damaging effect on the gene or gene product	PS3: Well-established in vitro or in vivo functional studies supportive of a damaging effect on the gene or gene product	NA
Segregation data	NA	NA	NA
Allelic data	PM3_1: For recessive disorders, detected in trans with a pathogenic variant – one observation (this study) of the variant in trans with other pathogenic variants	PM3_1: For recessive disorders, detected in trans with a pathogenic variant – one observation (this study) of the variant in trans with other pathogenic variants	PM3_PP: For recessive disorders, detected in homozygous state – one observation (this study)
Conclusion	***Pathogenic*** *ClinGen Bayesian: pathogenic (p = 1)*	***Pathogenic*** *ClinGen Bayesian: pathogenic (p = 1)*	***Pathogenic*** *ClinGen Bayesian: pathogenic (p = 0.999)*

Supplementary Table 5. Haplotype analysis of the recurrent *CEP78* deletion. Segregation analysis of 18 flanking single nucleotide polymorphisms (SNPs) revealed a common haplotype between the individuals carrying the deletion, with a minimal size of 1.9 Mb. The haplotype reconstruction includes SNP evaluation in the *CEP78* patient originally described (Sanchis-Juan et al., 2018) with the complex SV (deletion-inversion-deletion). Abbreviations used: Del1: deletion-inversion-deletion (deletion spanning exon 1-5 *CEP78*); wt: wild type; F: family.

**Marker**	**Position (hg38)**	**Av. Het**	**Allele UCSC**	**F1 II:1**	**F3 II:1**	**F3 I:2** (Mother)	**F3 I:1** (Father)	**SV Sanchis-Juan *et al.***
rs524314	chr9:75474883	0,481242	C	CC	CA	AA	CC	AA
rs1029037	chr9:76039060	0,499490	A	CC	AA	AA	CA	AA
rs9314838	chr9:76147202	0,499250	C	CC	CC	CC	CC	TT
rs631887	chr9:76497284	0,499990	C	CC	CT	CT	CT	TT
rs10156442	chr9:76591810	0,492679	G	GG	GA	GG	AA	AA
rs1953019	chr9:77150655	0,499539	A	AA	AC	CC	AA	CC
rs2026000	chr9:77720065	0,498673	A	**GG**	**G**G	**G**A	**G**G	**GG**
rs4877499	chr9:78233046 In deleted region	0,490231	G	NA	GG	CG	CC	NA
CEP78	chr9:78236065-78266994			Del1/Del1	Del1/ c.1449dup	c.1449dup/wt	Del1/ wt	Del1/ Del1
rs2521904	chr9:78713516	0,493247	G	**GG**	**G**G	**G**A	**G**G	**GG**
rs7869495	chr9:78929810	0,498982	G	**GG**	**G**C	**C**G	**G**C	**GG**
rs11792810	chr9:79255464	0,499609	C	**TT**	**T**T	**T**C	**T**T	**TT**
rs10780305	chr9:79363793	0,499977	G	**GG**	**G**G	**G**A	**G**G	**GG**
rs946806	chr9:79570457	0,498206	A	**AA**	**A**G	**G**G	**A**G	**AA**
rs7039267	chr9:79697131	0,495999	G	**AA**	**A**G	**G**G	**A**G	**AA**
rs7041989	chr9:79819028	0,482905	T	TT	TG	TT	TG	TT
rs2988072	chr9:79935341	0,483126	C	CC	CC	CC	CT	CC
rs7032321	chr9:79984278	0,487684	A	AA	AC	CC	AC	AA
rs12335799	chr9:81059207	0,484066	G	GG	CG	GG	CG	GG

Supplementary Table 6. Bioinformatics analysis of the breakpoints/junctions of the *CEP78* SVs identified in this study. The presence of microhomology at the breakpoints was assessed using a multiple sequence alignment between the junction fragment and the proximal and distal breakpoint regions, using ClustalO (https://www.ebi.ac.uk/Tools/msa/clustalo/; details in Supplementary Table 7). If both breakpoints of an SV overlap with a repetitive element, the consensus sequence was retrieved from UCSC and sequence identity between the repetitive elements was determined using BLAST2 (https://blast.ncbi.nlm.nih.gov/Blast.cgi?PAGE_TYPE=BlastSearch&BLAST_SPEC=blast2seq&LINK_LOC=align2seq). The presence of 40 previously described sequence motifs (Vissers et al., 2009) was investigated using Fuzznuc (http://www.bioinformatics.nl/cgi-bin/emboss/fuzznuc). The ability of DNA sequences to form non-B DNA conformations in the breakpoint regions was examined using several tools: GT repeats, forming left handed Z-DNA using non-B DNA motif search tool (nBMST, https://nonb-abcc.ncifcrf.gov/apps/nBMST/default/); direct, inverted and mirror repeats, forming slipped hairpin, cruciform and triplex structures, respectively, using nBMST, Quadruplex forming G-Rich Sequences (QGRS) using QGRS Mapper (Kikin, D’Antonio, & Bagga, 2006). All this data was

analyzed together in order to establish the most likely molecular mechanism underlying the SVs. Hg38 nomenclature is used. Breakpoint and junction numbering corresponds to the numbering on Supplementary Figure 10. Bp: base pair; BP: breakpoint; chr: chromosome; J: junction, kb: kilobase.

**SV**	Complex SV (J1)	Complex SV (J2)	Simple SV
**Deletion/duplication/Inversion**	Deletion - Inversion - Deletion	Deletion - Inversion - Deletion	Deletion
**Start (hg 38) chr9**	Deletion 1:78228782	Deletion 2:78234844	78096930
**End (hg 38) chr 9**	Deletion 1:78234546	Deletion 2:78244762	78331887
**Size (kb)**	5.7	9.9	234.9
**Consecutive microhomology (bp) at novel junction**	J1 (1-3): 2	J2 (2-4): 3	J1 (1-2): 4
**Repetitive elements intersecting the 5' breakpoint**	BP1: L1MB8	BP2: L2c, L1ME3Cz	BP1: /
**Number of sequence motifs**	2	4	0
**Number of non-B DNA conformation prediction motifs**	1	-	-
**Repetitive elements intersecting the 3' breakpoint**	BP3: L1ME3Cz	BP4: L1ME3Cz	BP2: AluJr4
**Number of sequence motifs**	4	5	4
**Number of non-B DNA conformation prediction motifs**	-	-	-
**Sequence identity between repetitive elements**	No significant similarity found	No significant similarity found	NA
**Potential molecular mechanism**	Most likely replicative	Most likely replicative	Most likely replicative

Supplementary Table 7. Microhomology at the SV junctions. For both SVs identified in this study, the presence of (consecutive) microhomology at the breakpoints was first assessed using a multiple sequence alignment between the junction fragments and the proximal (orange) and distal (grey) breakpoint regions (150 bp) using Clustal Omega (https://www.ebi.ac.uk/Tools/msa/clustalo/). Microhomology at the breakpoints was then indicated in bold text. Breakpoint and junction numbering corresponds to the numbering on Supplementary Figure 10.

**Complex SV: Junction 1**	PROXIMAL TGAGTGGATATTTTCTAGTGTAACATGTAATTGTGTTAATAATCTCTAAT<br>JUNCTION1 TGAGTGGATATTTTCTAGTGTAACATGTAATTGTGTTAATAATCTCTAAT<br>DISTAL TGTGCGACATGCTCAAATCTTAATATAGTAAACGCTTGTATATTCATAAA<br><br>PROXIMAL TGTATTTCTTTAGTTAATAGTTG**CC**CTAAAGCTTATGGTATTCATCTTAT<br>JUNCTION1 TGTATTTCTTTAGTTAATAGTTG**CC**ATTCCTGTTGAAGAGCTTTGTGTAA<br>DISTAL CCAGCTTAATAAATGAAACATTA**CC**ATTCCTGTTGAAGAGCTTTGTGTAA<br><br>PROXIMAL CAGAATCTACTTTAGATTTATACTAACTTCATTCCAGTGAAATATAGAAA<br>JUNCTION1 CCACCTTCCCAATCACGACTACTCTCTGACTCTGAGATAACTGCTCTTCT<br>DISTAL CCACCTTCCCAATCACGACTACTCTCTGACTCTGAGATAACTGCTCTTCT
**Complex SV: Junction 2**	PROXIMAL CATTTATTTTCCCTGTTGTTATATGGGAGTTCCATGTAGAAACATACAGT<br>JUNCTION2 CATTTATTTTCCCTGTTGTTATATGGGAGTTCCATGTAGAAACATACAGT<br>DISTAL TAAGTTTACATTTACCATGTCATTTTTAGTGACTCTTCAGGGTTCCATTA<br><br>PROXIMAL AATGTGTCTGTTTTTCTGTTGA**TGG**TAATTTAAATTGTTTCCAACTTTAC<br>JUNCTION2 AATGTGTCTGTTTTTCTGTTGA**TGG**CTTATCCGTTCCTATTAGATATATA<br>DISTAL TGTTAATATGCAATAGTACACT**TGG**CTTATCCGTTCCTATTAGATATATA<br><br>PROXIMAL GATTTCTTTGTTGCTCTATCCATTTTCTTTCTAGTCTCCAGCATCAATT<br>JUNCTION2 AGCTATTTCCAGTTTTTCATTGTTATAAACAGTACTTTGATACGTGTGT<br>DISTAL AGCTATTTCCAGTTTTTCATTGTTATAAACAGTACTTTGATACGTGTGT
**Simple SV: Junction**	PROXIMAL TCTGTGTTTCATTGACTAGCTGCCCATTAAATCATAGTTTGTCATTTACT<br>JUNCTION TCTGTGTTTCATTGACTAGCTGCCCATTAAATCATAGTTTGTCATTTACT<br>DISTAL CAGACTAGTCTTGAATTCCTGGCTGCAAGTGATCCTTTTGTCTTGGCCTC<br><br>PROXIMAL ACATGCTTTCCCTAGGTGTT**AGCC**TTAAATTGTGGCCAAGAATCATTCTA<br>JUNCTION ACATGCTTTCCCTAGGTGTT**AGCC**T**AGCC**ACTGCGTCTGGCTGAATGAAA<br>DISTAL CCAAAGTGCTGGGATTGCAGGCAAG**AGCC**ACTGCGTCTGGCTGAATGAAA<br><br>PROXIMAL CTTTACCAACTTTAAACTTTGACATCTTTAAGCATTTAAGAAAAATGCAA<br>JUNCTION AATCTTAATGTAGGTCACGTTGGTCTTTCTGAGTACCATAAACTGAGCTT<br>DISTAL AATCTTAATGTAGGTCACGTTGGTCTTTCTGAGTACCATAAACTGAGCTT

SUPPLEMENTARY REFERENCES

Abou Tayoun, A. N., Pesaran, T., DiStefano, M. T., Oza, A., Rehm, H. L., Biesecker, L. G., & Harrison, S. M. (2018). Recommendations for interpreting the loss of function PVS1 ACMG/AMP variant criterion. *Human Mutation*. https://doi.org/10.1002/humu.23626

Biesecker, L. G., Harrison, S. M., & ClinGen Sequence Variant Interpretation Working Group. (2018). The ACMG/AMP reputable source criteria for the interpretation of sequence variants. *Genetics in Medicine : Official Journal of the American College of Medical Genetics*, *20*(12), 1687–1688. https://doi.org/10.1038/gim.2018.42

Ellard, S., Baple, E. L., Berry, I., Forrester, N., Turnbull, C., Owens, M., … McMullan, D. J. (2019). ACGS Best Practice Guidelines for Variant Classification 2019. *Association for Clinical Genetic Science*.

Ellard, S., Baple, E. L., Owens, M., Eccles, D. M., Abbs, S., & Zandra, C. (2020). ACGS Best Practice Guidelines for Variant Classification 2020 v4.01. *Association for Clinical Genetic Science*.

Kikin, O., D'Antonio, L., & Bagga, P. S. (2006). QGRS Mapper: A web-based server for predicting G-quadruplexes in nucleotide sequences. *Nucleic Acids Research*. https://doi.org/10.1093/nar/gkl253

Nykamp, K., Anderson, M., Powers, M., Garcia, J., Herrera, B., Ho, Y. Y., … Topper, S. (2017). Sherloc: A comprehensive refinement of the ACMG-AMP variant classification criteria. *Genetics in Medicine*. https://doi.org/10.1038/gim.2017.37

Sanchis-Juan, A., Stephens, J., French, C. E., Gleadall, N., Mégy, K., Penkett, C., … Carss, K. J. (2018). Complex structural variants in Mendelian disorders: identification and breakpoint resolution using short- and long-read genome sequencing. *Genome Medicine*, *10*(1), 95. https://doi.org/10.1186/s13073-018-0606-6

Vissers, L. E. L. M., Bhatt, S. S., Janssen, I. M., Xia, Z., Lalani, S. R., Pfundt, R., … Stankiewicz, P. (2009). Rare pathogenic microdeletions and tandem duplications are microhomology-mediated and stimulated by local genomic architecture. *Human Molecular Genetics*. https://doi.org/10.1093/hmg/ddp306
